# Strong Evidence for an Intraspecific Metabolic Scaling Coefficient Near 0.89 in Fish

**DOI:** 10.3389/fphys.2019.01166

**Published:** 2019-09-20

**Authors:** Christopher L. Jerde, Krista Kraskura, Erika J. Eliason, Samantha R. Csik, Adrian C. Stier, Mark L. Taper

**Affiliations:** ^1^Marine Science Institute, University of California, Santa Barbara, Santa Barbara, CA, United States; ^2^Department of Ecology, Evolution, and Marine Biology, University of California, Santa Barbara, Santa Barbara, CA, United States; ^3^Department of Ecology, Montana State University, Bozeman, MT, United States; ^4^Department of Biology, University of Florida, Gainesville, FL, United States

**Keywords:** likelihood, evidence functions, SIC, standard metabolic rate, mixed effects models, metabolic scaling, evidentialist statistics

## Abstract

As an example of applying the evidential approach to statistical inference, we address one of the longest standing controversies in ecology, the evidence for, or against, a universal metabolic scaling relationship between metabolic rate and body mass. Using fish as our study taxa, we curated 25 studies with measurements of standard metabolic rate, temperature, and mass, with 55 independent trials and across 16 fish species and confronted this data with flexible random effects models. To quantify the body mass – metabolic rate relationship, we perform model selection using the Schwarz Information Criteria (ΔSIC), an established evidence function. Further, we formulate and justify the use of ΔSIC intervals to delineate the values of the metabolic scaling relationship that should be retained for further consideration. We found strong evidence for a metabolic scaling coefficient of 0.89 with a ΔSIC interval spanning 0.82 to 0.99, implying that mechanistically derived coefficients of 0.67, 0.75, and 1, are not supported by the data. Model selection supports the use of a random intercepts and random slopes by species, consistent with the idea that other factors, such as taxonomy or ecological or lifestyle characteristics, may be critical for discerning the underlying process giving rise to the data. The evidentialist framework applied here, allows for further refinement given additional data and more complex models.

## Introduction

One of most contentious controversies in ecology is the scaling relationship between an organism’s body mass and metabolic rate (Agutter and Wheatley, 2004; Isaac and Carbone, 2010; Glazier, 2018). Kleiber (1932) popularized the idea that contrary to a century of theory, a mammal’s metabolic rate (*MR*) scales with body mass (*BM*) not as a power law with an exponent of *β* = 0.67, but as a power law with an exponent of *β* = 0.75. This relationship takes the form

(1) ln⁡(M⁢R)=β×ln⁡(B⁢M)+c

where *β* is the scaling relationship and *c* is an intercept from a liner regression. As a cornerstone of the metabolic theory of ecology (Brown et al., 2004), this 0.75 scaling relationship is used to link individual physiology to the observed patterns of communities and energy flows across landscapes. The 0.75 value has been mechanistically justified through hypotheses that maximize energy delivery to tissue in animals (West et al., 1997) and from xylem and phloem networks that transport water and nutrients in plants (Enquist and Niklas, 2001). However, the universality of the 0.75 value is eagerly disputed, with alternative hypotheses and empirical studies putting the scaling relationship commonly between 0.5 and 1 (Bokma, 2004; Glazier, 2018).

Intraspecific (within species) scaling has been proposed to differ from interspecific (between species) scaling and also different mechanisms may be responsible for different scaling relationships. Metabolic rates vary 2–3 fold across individuals of the same population and this variation is repeatable (Burton et al., 2011; Norin and Malte, 2011; Boldsen et al., 2013). Intraspecific scaling has received less attention than interspecific scaling, while even fewer studies have investigated scaling relationships within each tested individual as it grows (but see Norin and Gamperl, 2018). Both intraspecific and interspecific scaling are critical for linking species physiology to projections of population abundance (Kooijman, 1993) and predicting the impacts of climate change on species distributions (Sunday et al., 2010; Lindmark et al., 2018).

While the implications of deviations from the 0.75 scaling exponent are large, there is limited data available to accurately estimate the exponent. This is because measuring the metabolic rate of an individual is not a trivial experiment, let alone across a 10-fold range of body sizes from a population, at different temperatures, and/or across species (Lighton, 2018). To date, most studies have relied on either a limited study design (one species, many individuals, with fixed treatments of temperature; Table 1) or meta-analysis of mean metabolic rate data across studies using variable methods of measurement (Glazier, 2005). While the former can suffer from insufficient sample sizes, measurement error, and unaccounted for factors influencing the general relationship, the latter treats all studies equally and both approaches have ultimately been inconclusive as to the evidence supporting or refuting competing hypotheses (Glazier, 2018) with some concluding there is not a universal scaling constant (Bokma, 2004).

**TABLE 1:** Overview of metabolic studies.

**Citation**	**Species**	***n***	**Temp.**	**(Min, Max)**	**Regression**	**Trial**
			**(°C)**	**weight (g)**	**coefficient $\hat{\beta }$, (SE)**	
(1) Norin and Gamperl, 2018	Cunner (*Tautogoabrus adsperus*)	68	15	0.45, 4.61	0.92 (0.035)	1
Norin and Gamperl, 2018	Cunner (*Tautogoabrus adsperus*)	68	15	0.97, 7.94	0.98 (0.028)	2
Norin and Gamperl, 2018	Cunner (*Tautogoabrus adsperus*)	68	15	1.24, 13.2	0.89 (0.024)	3
Norin and Gamperl, 2018	Cunner (*Tautogoabrus adsperus*)	68	15	1.56, 15.56	0.83 (0.024)	4
Norin and Gamperl, 2018	Cunner (*Tautogoabrus adsperus*)	68	15	1.71, 19.46	0.79 (0.026)	5
(2) Auer et al., 2015	Brown Trout (*Salmo trutta*)	120	11.5	5.48, 16.12	0.61 (0.068)	6
(3) Behrens et al., 2017	Round Goby (*Neogobius melanostomus*)	8	15–17	43, 73	1.031 (0.24)	7
Behrens et al., 2017	Round Goby (*Neogobius melanostomus*)	8	15–17	35, 78	1.38 (0.16)	8
Behrens et al., 2017	Round Goby (*Neogobius melanostomus*)	8	15–17	36, 72	0.9 (0.17)	9
(4) Killen, 2014	Common Minnow (*Phoxinus phoxinus*)	13	10	0.72, 2.03	0.78 (0.27)	10
(5) Norin and Clark, 2017	Barramundi (*Lates calcarifer*)	24	29	23.1, 37.6	0.91 (0.17)	11
(6) McLean et al., 2018	Common Minnow (*Phoxinus phoxinus*)	123	13	0.68, 7.44	0.72 (0.07)	12
(7) Boldsen et al., 2013	European Eel (*Anguilla anguilla*)	24	20	184, 507	1.44 (0.25)	13
Boldsen et al., 2013	European Eel (*Anguilla anguilla*)	24	20	171, 504	1.05 (0.21)	14
(8) Kunz et al., 2016	Polar Cod (*Boreogadus saida*)	5	0	18.5, 27.4	0.81 (0.35)	15
Kunz et al., 2016	Polar Cod (*Boreogadus saida*)	5	3	16.1, 48.6	0.96 (0.1)	16
Kunz et al., 2016	Polar Cod (*Boreogadus saida*)	5	6	22.7, 32.8	1.06 (0.41)	17
Kunz et al., 2016	Polar Cod (*Boreogadus saida*)	6	8	11.4, 29.1	1.03 (0.3)	18
Kunz et al., 2016	Atlantic cod (*Gadus morhua*)	12	3	21.2, 105	0.97 (0.15)	19
Kunz et al., 2016	Atlantic cod (*Gadus morhua*)	10	8	45.7, 173.6	0.9 (0.15)	20
Kunz et al., 2016	Atlantic cod (*Gadus morhua*)	7	12	54.5, 149.1	1.1 (0.13)	21
Kunz et al., 2016	Atlantic cod (*Gadus morhua*)	5	16	83.2, 156.2	1.05 (0.18)	22
(9) Norin et al., 2016	Barramundi (*Lates calcarifer*)	60	29	23.08, 48.96	1.03 (0.13)	23
(10) Collins et al., 2016	Barramundi (*Lates calcarifer*)	20	30	153.9, 453.7	1.07 (0.14)	24
Collins et al., 2016	Barramundi (*Lates calcarifer*)	20	30	196.3, 390	1.19 (0.28)	25
(11) Khan et al., 2014	Hapuku Wreckfish (*Polyprion oxygeneios*)	8	12	88.2, 131.2	0.93 (0.45)	26
Khan et al., 2014	Hapuku Wreckfish (*Polyprion oxygeneios*)	8	15	105.3, 164.5	0.64 (0.44)	27
Khan et al., 2014	Hapuku Wreckfish (*Polyprion oxygeneios*)	8	18	146.1, 203.2	−0.21(0.48)	28
Khan et al., 2014	Hapuku Wreckfish (*Polyprion oxygeneios*)	8	21	130.3, 188.6	0.61 (0.26)	29
Khan et al., 2014	Hapuku Wreckfish (*Polyprion oxygeneios*)	8	24	97.7, 131.6	1.2 (0.36)	30
(12) Khan et al., 2018a	Rainbow Trout (*Oncorhynchus mykiss*)	16	16	69.9, 120.2	0.87 (0.32)	31
(13) Khan et al., 2018b	Atlantic Salmon (*Salmo salar*)	25	14	39.1, 70.7	0.57 (0.22)	32
(14) Khan et al., 2015	Hapuku Wreckfish (*Polyprion oxygeneios*)	12	15	196.1, 324	0.84 (0.15)	33
(15) Khan et al., 2015	Hapuku Wreckfish (*Polyprion oxygeneios*)	12	21	114.5, 191	0.6 (0.2)	34
(16) Cooper et al., 2018	Three Spine Stickleback (*Gasterosteus aculeatus*)	31	12	0.46, 1.19	1.43 (0.39)	35
(17) McArley et al., 2017	Common Triplefin (*Forsterygion lapillum*)	20	15	1.59, 3.38	0.67 (0.19)	36
McArley et al., 2017	Common Triplefin (*Forsterygion lapillum*)	20	18	1.52, 3.81	0.82 (0.19)	37
McArley et al., 2017	Common Triplefin (*Forsterygion lapillum*)	23	21	1.54, 3.42	0.78 (0.15)	38
(18) McArley et al., 2018	Twister (*Bellapiscis medius*)	10	21	1.53, 3.98	0.94 (0.1)	39
McArley et al., 2018	Common Triplefin (*Forsterygion lapillum*)	10	21	1.27, 2.97	0.45 (0.16)	40
(19) Eliason et al., 2007	Rainbow Trout (*Oncorhynchus mykiss*)	24	8–14	381, 652.7	0.64 (0.74)	41
Eliason et al., 2007	Rainbow Trout (*Oncorhynchus mykiss*)	5	11–16	564.8, 3233.6	1.33 (0.3)	42
(20) Norin and Malte, 2011	Brown Trout (*Salmo trutta*)	33	15	20.7, 45.7	1.5 (0.18)	43
Norin and Malte, 2011	Brown Trout (*Salmo trutta*)	33	15	27.4, 55.1	1.19 (0.14)	44
Norin and Malte, 2011	Brown Trout (*Salmo trutta*)	33	15	37.7, 64.9	0.98 (0.18)	45
Norin and Malte, 2011	Brown Trout (*Salmo trutta*)	33	15	38.4, 68.2	1.11 (0.17)	46
(21) Norin and Malte, 2012	Brown Trout (*Salmo trutta*)	66	15	20.5, 57.7	1.09 (0.094)	47
(22) Nadler et al., 2016	Blue Green Puller (*Chromis viridis*)	16	29	1.3, 2.1	0.63 (0.3)	48
(23) Collins et al., 2013	Barramundi (*Lates calcarifer*)	9	26	172, 205	0.18 (1.03)	49
Collins et al., 2013	Barramundi (*Lates calcarifer*)	10	26	186, 221	2.06 (1.28)	50
Collins et al., 2013	Barramundi (*Lates calcarifer*)	10	26	169, 215	1.49 (0.78)	51
Collins et al., 2013	Barramundi (*Lates calcarifer*)	11	26	139, 244	0.65 (0.43)	52
Collins et al., 2013	Barramundi (*Lates calcarifer*)	9	26	184, 233	0.71 (0.54)	53
(24) Zhang et al., 2017	European Sea Bass (*Dicentrarchus labrax*)	11	16.5	48.1, 100.7	1.01 (0.18)	54
(25) Zhang et al., 2016	Atlantic Salmon (*Salmo salar*)	87	12	23.4, 57	1.15 (0.11)	55

In this *Frontiers* Research Topic devoted to evidential statistics, model identification, and science, multiple contributions (Dennis et al., 2019) show how standard statistical approaches (such as Fisherian significant tests, Neyman-Pearson hypothesis testing, Akaike Information Criterion for multi-model inference) are misleading when models used for inference are misspecified. Model misspecification is arguably the case for most analyses, including ours, that seek to evaluate the evidence of a universal scaling relationship across a broad range of fish species, at different temperatures, and using studies, that have reliable data, but that were not necessarily designed to have a large range of body masses across which to regress metabolic rate. Here we demonstrate how an evidentialist approach can be applied to gain novel insight to the question, “What is the evidence for an intraspecific universal scaling relationship between fish body mass and metabolic rate?”

### Scaling Relationships as Hypotheses for Fish

Multiple mechanisms have been put forth to justify *β* = 0.67, 0.75, and 1 scaling relationships. If the primary limitation for resources or waste removal is transport of chemicals across surfaces, then metabolic rate is predicted to scale with surface area with a relationship of 0.67. For example, Killen et al. (2010) found that highly active, pelagic fishes had a scaling relationship of 0.7 (SE 0.04), close to 0.67, which they attributed to a constraint in oxygen or fuel acquisition or waste removal across surface areas in these metabolically active fishes. However, the 0.67 scaling exponent is more commonly found in endotherms, mammals and birds, but rarely in ectotherms (White and Seymour, 2003; White et al., 2005).

If metabolic rate is primarily limited by the fractal nature of distribution networks (e.g., the internal transport networks of resources and wastes), then a scaling relationship of 0.75 is predicted (West et al., 1997). Previous synthesis of teleost fish found a scaling relationship of 0.79 (SE 0.11) (Clarke and Johnston, 1999), and with sufficient variability as to not exclude the 0.75 value used by Metabolic Theory of Ecology to explain broad ecological patterns (Brown et al., 2004). Similarly, Moses et al. (2008) showed metabolic scaling during ontogeny for seven fish species was 0.78 (SE 0.02), with some variability in slope estimates between species.

Metabolic rate is predicted to be directly proportional to body size (i.e., *β* = 1) when maintenance and routine activity costs are low and these demands can easily be met by both surface area and internal transport mechanisms. In the case of less active fish or those occupying deeper waters, individual metabolism has been demonstrated to scale nearly proportionally to body mass [i.e., scaling exponents approach 1 (Killen et al., 2010)].

Two more recent hypotheses work with the common observation that scaling exponents vary (e.g., Glazier, 2018). The metabolic-level boundaries (MLB) hypothesis of scaling (Glazier, 2008) states that any observed scaling exponent varies within the limits of 0.67 and 1, representing whether the mechanisms or processes that underlay the scaling relationship are predominantly limited by surface area constraints on fluxes of resources, waste and heat (0.67; e.g., gill surface area, internal transport limitation) or by volume (mass) constraints on energy demand or production of tissue (1; assuming energy demand is proportional to tissue size). Therefore, MLB also provides an explanation to variable scaling exponents of animals at different physiological states, or routine requirements. Alternatively, Dynamic Energy Budget (DEB) theory (Kooijman, 1993) provides a more recent approach predicting metabolic scaling relationships in all species irrespective to taxonomical classification; this approach is based solely on physical principles, and uses storage of nutrients (reserves increase with increasing structure) as a central mechanism explaining both intra- and inter species-specific scaling relationships (Maino et al., 2014). While both MLB and DEB would seemingly make the case that a universal scaling exponent does not exist and should consequently not be expected, they do not preclude a mean universal scaling exponent.

### Temperature and Other Factors

Temperature plays a critical role regulating individual metabolic rate in ectotherms such as fishes (Brett and Glass, 1973; Johnston and Dunn, 1987). The effects of temperature on the metabolic scaling relationship has been studied mechanistically (Gillooly et al., 2001) with syntheses showing low temperature sensitivity from resting measures of metabolism and a consistent metabolic scaling relationship (Clarke and Johnston, 1999, but see Lindmark et al., 2018).

Numerous ecological, physiological and lifestyle characteristics can influence metabolic rate and potentially affect scaling relationships. Metabolic rate in ectotherms is strongly dependent on physical and chemical characteristics of the water they live in, and consequently shows context-dependent variation (Killen et al., 2016). Therefore, habitat (abiotic factors), predation risk, activity level, food availability, and social status and behavioral traits, all can affect metabolic rates (for a review on variation of fish standard metabolic rate (SMR), see Metcalfe et al., 2016), thus also likely scaling parameters, especially intercept. For example, food availability affects growth rates and is linked to SMR variation in fish (Killen, 2014; Auer et al., 2015). Auer et al. (2018) demonstrated a strong dependence of SMR on individual ecology underlined by predation level, reproductive age and investment, longevity, and maximum body size (life-history traits). Many of these factors vary in unique combinations across populations of the same species (Eliason et al., 2011; Auer et al., 2018), therefore even within species we may expect variation in metabolic rate and its dependence on size.

### Sources of Uncertainty and Measurement Error

Misspecification is a model that does not account for variables (i.e., temperature) or structural forms (i.e., random effects) that can lead to biased coefficients, misleading error terms, and unlimitedly wrong inferences about the generating process giving rise to the data (White, 1982). While temperature has been identified as a critical covariate for fish (Brett and Glass, 1973), other necessary covariates are less clear, but one should assume there is likely something missing. Additionally, as any model expands its inferential breadth beyond a single species, the model will become more complex either by adding fixed effects to measure species-level coefficients or by treating species as a random effect of the model from which to make inference across all fish. The advantage of using random effects to make broader inferences has been well recognized across ecology (Bolker et al., 2009). Such is the case when making population level inferences in resource selection functions from location data from multiple individuals (Gillies et al., 2006). However, more information on the species level traits may lead to better models and improved inferences.

The quality of the data will also impact inferences. One known source of uncertainty is measurement error – that is the errant measurement of observations, such as body mass. Farrell-Gray and Gotelli (2005) clearly showed that errant measurement of the predictor variable of mass biased the estimated slope parameter of the metabolic relationship and speculated that allometric exponents lower than 0.75 may be due simply to measurement error. The magnitude of the effect of measurement error in a predictor variable on the estimated slope of a linear regression is well known: $E\,\left(\hat{\beta }\right)=\lambda \,\beta$, where λ, the reliability coefficient, is the proportion of variation in the predictor variable not due to measurement error (Taper and Marquet, 1996; Cheng and Van Ness, 1999). The lower reliability the more biased the estimate. In Box 1, we evaluated the influence of measurement error for California spiny lobster (*Panulirus interruptus*), albeit not a fish, but find very little evidence for any bias due to measurement error from retained residual water. We assume going forward, that for fish, measurement error is not biasing our parameter estimates.

**BOX 1. Measurement error in body mass of lobsters.**

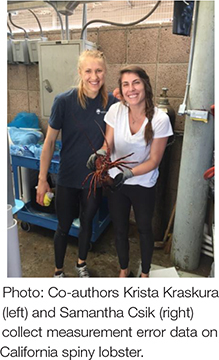

California spiny lobster (*Panulirus interruptus*) is commercially highly valued, and is ecologically important having a large effect on trophic dynamics and ecosystem resilience in kelp forests and rocky reef beds (Dunn et al., 2017; Caselle et al., 2018). Metabolic rate in ectotherms directly depends on animal’s body size and temperature and represents the pace of nearly all biological processes. Meanwhile, MR varies within and among individuals (Glazier, 2005; White and Kearney, 2013; Norin and Gamperl, 2018). Lobsters are cumbersome to weigh, thus making them a good candidate to explore how measurement error in body mass may affect metabolic scaling.

Lobsters were collected by divers via SCUBA (CDFW Scientific Collection Permit #13746) and maintained in 110-gallon flow-through seawater tanks divided in half with perforated PVC. One individual was held in each half tank (24”L × 30”W × 18”H), and provided with 10” PVC cut in half to create structure and habitat. Lobsters were fed mussels (*Mytilus* spp.) *ad libitum* when not being used in respirometry trials. Animals were held at ambient temperatures and exposed to natural light.

To estimate measurement error, 45 lobsters were weighed three consecutive times. Before weighing, individual’s dorsal side and tail were dried with a microfiber towel. The mass was measured to the nearest gram. Lobsters were fully submerged between repeat trials.

From the log transformed mass measurements (*n* = 45), the pooled error variance is 1.2 × 10^–5^ (SD 0.0035). We regressed the within individual standard deviation against the mean log(weight), but the slope was not different from zero and Levene’s test did not indicate there is any heterogeneity. From inspection of the pooled error variance, there is very little variability in the individual measurements of body mass. Furthermore, regression revealed no trend in error variance as function of mean body mass.

For six lobsters, ranging in body mass from 175 to 2426 g, we conducted a more thorough drying by carefully removing water from the leg joints, carapace, and underside of the lobster abdomen, spending approximately double the time drying than the standard protocol called for. We regressed the mean log(weight) against the thoroughly dried log (weight) for the six lobsters. Expectedly, the intercept (0.05, SE 0.008) and slope (0.994, SE 0.0001) were statistically significant (*p* < 0.001), but the residual standard was very small (0.0033), indicating that *measurement error in mass is negligible.* Thus, for all regressions with log(weight) as a predictor variable, the reliability ratio will be effectively 1 and there will be no bias in estimated slopes due to measurement error.

Measurement error in the response variable, metabolic rate in our study, leads to greater residual variability but no bias in the slope parameter. However, the added variability in the residual error can inflate our uncertainty surrounding the slope parameter leaving us unable to distinguish between potential hypotheses (competing models). Metabolic rate (MR) represents a sum of all chemical reactions that take place in an organism, and this may change drastically upon any intrinsic and extrinsic change, e.g., spontaneous activity, physiological disturbance, feeding, and even just circadian rhythms. To refine how MR varies as function of mass, it is a necessity that the data originate from animals at the same physiological states. Standard metabolic rate, SMR is defined as the subsistence metabolism to support body maintenance in a post-absorptive, resting state under thermally acclimated conditions (Chabot et al., 2016). True SMR is often impractical and challenging to measure in fishes, and so data often reflects routine metabolic rates, which alternatively may be perceived as a measurement error (in the response, Y axis) around individual SMR, which increases variability but does not bias the slope parameter. With a goal to minimize such variation, we developed specific experimental criteria for data to be included (see section “Data”). For a good overview of methods and approaches to metabolic scaling in animals see White and Kearney (2014).

## Materials and Methods

The general approach we implemented for this study is to: (1) include reliably collected SMR data based on recently published studies (200-present), (2) apply flexible, mixed effect linear models, and (3) employ an evidence function, the Schwarz Information Criterion (SIC), to evaluate the evidence for specified mechanistic hypotheses of the scaling relationship of *β* = 0.67, 0.75, 1, and *β* as an estimated, free parameter $\left(\hat{\beta }\right)$.

### Data

The approaches and technology used to measure fish metabolism have become more accurate, precise, and robust within the last 20 years (Nelson, 2016). We curated published data sets of individual fish metabolism comprised of fish that were: 1) post larval life stages, 2) in a post-absorptive state, meaning they were unfed for a minimum of 20 h prior to taking metabolic rate measurements, 3) with overnight metabolic rates (>12 h of automatic measurement), 4) with an acclimated water temperature for at least 7 days prior to the experiment, and 5) were at calm resting states. Studies where species were manipulated, such as treatments to measure the effects of starvation on SMR, or where the study’s authors noted substantial spontaneous activity were not included. Further, we ensured robust data analysis methods were used to calculate SMR following Chabot et al. (2016) and where SMR was measured at ecologically relevant temperature ranges for each species. Studies were not considered if they included surgical manipulations with the exception of non-invasive tagging (e.g., using passive integrated transponder (PIT) and visible implant elastomer tags). Data were not included if the study’s methods lacked sufficient detail in any of the above criteria, the Supplementary Data online were not clear, or appeared to contain errors. All fish included were lab residents for at least 2 weeks before the SMR measurement took place.

**FIGURE 1:**
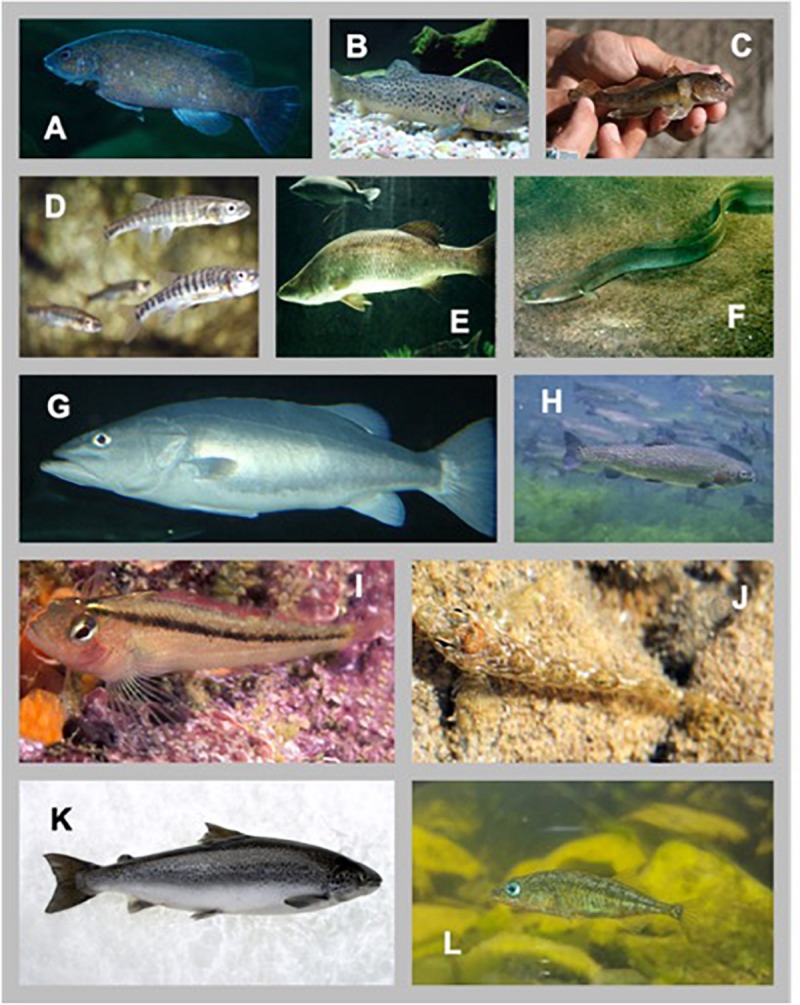
Diversity of species used in this study. **(A)** Cunner (https://commons.wikimedia.org/wiki/File:Cunner.jpg; to Flickr, by Vhorvat), **(B)** Brown Trout (https://commons.wikimedia.org/wiki/File:Brown_trout.JPG; Zouavman Le Zouave), **(C)** Round Goby (https://www.michigan.gov/invasives/0,5664,7-324-68002_73845-368437–,00.html; David Copplestone), **(D)** Common Minnow (Subaqueous Vltava, Prague 2011, Czechia; Provided by Karelj), **(E)** Barramundi (https://commons.wikimedia.org/wiki/File: Barramundi.jpg provided by Nick Thorne), **(F)** European Eel (https://commons.wikimedia.org/wiki/File:Anguilla_anguilla.jpg; GerardM), **(G)** Hapuku Wreckfish (https://commons.wikimedia.org/wiki/File:Hapuka.jpg; Nholtzha), **(H)** Rainbow Trout (https://digitalmedia.fws.gov/digital/collection/natdiglib/id/2151 Eric Engbretson), **(I)** Common Triplefin (https://commons. wikimedia.org/wiki/File:Forsterygion_lapillum_(Common_triplefin).jpg; Ian Skipworth), **(J)** Twister (https://commons.wikimedia.org/wiki/File:Bellapiscis_ medius_2.jpg; A.C. Tatarinov), **(K)** Atlantic Salmon (https://commons. wikimedia.org/wiki/File:CSIRO_ScienceImage_8062_Atlantic_salmon.jpg; Peter Whyte, CSIRO), **(L)** Three-spined Stickleback (https://commons. wikimedia.org/wiki/File:Three-spined_Stickleback_(Gasterosteus_aculeatus)_ at_the_Palo_Alto_Junior_Museum_and_Zoo.jpg; Evan Baldonado/ AquariumKids.com).

Our database includes 25 studies, with 55 independent trials, across 16 fishes (Figure 1). Table 1 details the sources of the data, species, trials identification, temperature under which the SMR measurements were collected, and sample sizes per trial. A total of *n* = 1456 observations are used in the study. Some studies where not designed or conducted to estimate the scaling relationship between individual fish SMR and body mass – a notable point we will return to in later sections.

### Models

#### Linear Regression

Each trial (Table 1; *n* = 55) is an experiment of the metabolic scaling relationship of SMR to body mass. We applied linear regression to the log transformed SMR and body mass data for each trial. Because some of these studies were not designed to test this relationship, we expect the regression slope estimates to be variable and have large standard errors for those data sets with low sample size. Additionally, it is recommended to have a 4 to 10-fold range of fish body mass, but many trials and studies do not meet this recommendation. However, the data in totality has a range from 0.45 to 3233.6 g. We expect the distribution of slopes from trials to largely mirror the results found by Clarke and Johnston (1999).

#### Linear Mixed Effects Models

Using the lme4 package in the R statistical programing language (Bates et al., 2015), we tested four unique suites of model forms with combinations of fixed and random effects. For all models we included temperature (but see Box 5) and body mass as a fixed effect, and we treated trials within species as a nested effect. The first model suite allows intercepts to randomly vary among species. The second model suite, has fixed intercepts for each species with common slope, but does not assume a normal distribution of species’ intercepts. With 16 unique species, this second approach adds significantly more parameters to estimate, but allows for inferential insights into the differences between species. The third model suite uses a random slope and random intercept by species. The correlation between the slope and intercept is estimated and not assumed to be independent. The fourth model suite uses a random slope with estimated intercepts for each species. The random slopes are interpreted as by-species deviations from the fixed effect slope.

For each of the four approaches, we evaluate the fixed effect slope of body mass as a free parameter and then constrained the slope to equal each of our underlying mechanistic hypotheses of 0.67, 0.75, and 1.

### Analysis

All models were fit using Maximum Likelihood Estimation (MLE) and all analyses were conducted in the R statistical programing language (R Core Team, 2015).

#### Strategy of Scientific Inference and Statistical Tactics

Classical hypothesis testing has been the backbone of scientific inference for nearly a century. Both the Fisherian and the Neyman-Pearson variants of hypothesis testing turn on the axle of a counterfactual argument. The argument stripped of probabilistic uncertainty runs like this: If we assume a particular model (generally called the null) is true then we can predict that a specific pattern should occur in our data. If the predicted pattern does not occur, then the null hypothesis cannot be true and something else must be.

This argument has worked well for science in tightly controlled situations where the predicted patterns are clear and the nature of the “something else” is unequivocal. But in more open situations, with more experiments, more models, more questions and variable amounts of data, the chain of hypotheses (multiple models) becomes harder to follow and the statistical adjustments required to maintain even the illusion of control of error rates become more cumbersome. Paradoxically, considering more models and asking more questions makes it harder to find support for any model or to answer any question.

One common approach to multimodal inference is the application of information criterion (Burnham and Anderson, 2004). Akaike’s Information Criterion (AIC) is one such inductive inferential approach that is both widely recognized and applied (Akaike, 1981). The appeal of such an approach is to simultaneously assess competing hypotheses based on how well the models perform relative to each other through the likelihood function, but then discount the potential overfitting of models that have a large number of parameters.

User-defined thresholds demark ΔAIC values that constitute weak, strong, or very strong evidence for one model over the other. If parameters are estimated, the likelihood becomes a biased estimate of how close a model is to the generating process. The more parameters estimated, the greater this over optimism. Akaike (1973) initiated the use and study of information criteria, which correct for this bias. Information criteria have been enormously useful in analyzing biological data (see Burnham and Anderson, 2002). Many information criteria (the consistent criteria) fully meet all the criteria listed in Box 2 and are evidence functions.

**BOX 2. What is an evidence function?**

Evidence functions are based on nine desiderata (i.e., something that is desired or wanted) for statistical and philosophical properties with desirable and meaningful characteristics for scientific applications (Lele, 2004; Taper and Lele, 2011; Taper and Ponciano, 2016). Here, we attempt to translate those desired properties (D0 to D8) for scientists with emphasis on implications to applications.

D0: Evidence is measurable, does not require information about beliefs, and is made from confronting at least two models that represent scientific hypotheses with the data.
D1: Evidence functions measure how possible data under each model (at least two) match or are comparable to the observed data. Neither model may completely describe the process that generated the observed data, but the function can discriminate if one of the models is more likely to have generated the observed data.
D2: Evidence is continuous from virtually none to very strong, and measuring evidence should likewise be a continuous and not have a threshold like using α levels for hypothesis testing.
D3: Evidence must be arrived at in a reproducible way. If I do not describe processes by which I arrive at a conclusion, then it becomes difficult for someone else to follow the logic to get to that conclusion or challenge the underlying approach.
D4: Personal opinions, beliefs, or intentions cannot influence the evidence function in a hidden way and the process should be accessible to everybody. If a broader scientific audience does not understand what constitutes evidence, then the function cannot be used as evidence.
D5: Evidence functions do not change person to person (in contrast to Bayesian approaches with different personal priors).
D6: Evidence does not need to come from a single critical test (experiment). Evidence functions should have an explicit way of combining data sets to confront hypotheses and the process should be inherently dynamic with reevaluation as more data or better data are collected.
D7: The evidence should not change depending on the scale the data was collected and analyzed. Nor should evidence be sensitive on transformation of parameters. To give an example related to the metabolic scaling relationship research, if we allowed the appearance of plots to be evidence for the slope, then we could change our evidence by making one plot with one *x*-axis scale and another plot with different scale. One of the interests of this paper is how much difference there is among species in β. It should not make a difference to the evidence if this dispersion is parameterized as a variance or as a standard deviation.
D8: More data results in better inferences, but will only be as good as the completeness of the models/hypotheses tested. The model selected in any given analysis will, with more and more data collected, be the model closest to describing the process from which the data are observed. You can do no better in understanding the underlying process than the models contained within your suite of models evaluated.

Evidence for one model over another is a function of the estimated relative discrepancy of any two models from the generating process and is measured by evidence functions. Evidence functions (Box 2) can take many forms (see Lele (2004), and Taper and Lele (2011) for technical and philosophical discussions, and Taper and Ponciano (2016) for a more general discussion). The Schwarz Information Criterion (SIC) often referred to as the Bayesian Information Criterion (BIC), when used to compare differences between competing models (ΔSIC) is an evidence function (Dennis et al., 2019). Similar to AIC, the SIC (Eq. 2) uses the maximum likelihood function (*L*) to evaluate the fit of the model to the data and uses a function of the amount of data (*n*) and the number of parameters (*k*) to penalize for overfitting (Burnham and Anderson, 2004).


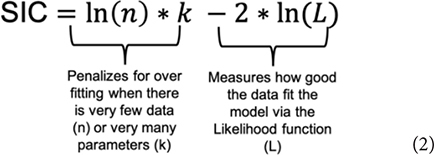


The SIC penalizes for model complexity more heavily than AIC and the error properties are aligned with the concept of evidence functions, whereas the AIC error properties are not (Dennis et al. this research topic). SIC is also commonly available in R packages (named the BIC). The criterion (Eq. 2) can be derived either in a Bayesian context (Schwarz, 1978) or in a frequentist context (Nishii, 1988) We adopt the SIC terminology throughout for model selection and evaluation of parameter uncertainty using ΔSIC intervals to avoid confusion of the evidentialist approach with Bayesian analysis and inference. The model with the lowest value of SIC is considered the best model and the evidence function, ΔSIC_ij_, is the pairwise difference formed by subtracting the SIC of a reference model i from the SIC of a competing model j. As an evidence function, ΔSIC_ij_ is continuous from negative infinity to infinity with the strength of evidence for the reference model over the competing model growing larger as the ΔSIC becomes positive and large. Commonly, when information criteria are used for model selection, the model in the model set with the lowest IC value is used as the reference model, and all ΔIC are therefore positive.

Given the hierarchical nature of mixed models several alternative effective sample sizes can be calculated (Jones, 2011); these methods adjust the sample size (*n*), used in the SIC calculation (Eq. 2) to the effective samples size to account for assumptions of non-independence in data. Which is most appropriate depends on the level in the hierarchy of inferential interest. Because the parameter of primary interest in this study is the fixed effect of body mass, the total sample size is the correct effective sample size to use (Lorah and Womack, 2019).

Instead of attempting to reject false models, the evidential approach seeks to assess which models are closer to the unknown natural generating process than other competing models. The support for one model does not in itself diminish support for other models. However, scientists may find themselves in the situation where several distinct models appear nearly as good. Given the data in hand, the scientist cannot strongly differentiate between the models in this set. In this case, all of these models should be retained in the scientist’s thinking.

#### ΔSIC Intervals

SIC values can also be used to define uncertainty surrounding a parameter estimate – thus linking model selection to measures of uncertainty directly through the use of ΔSIC. Discussion of evidential intervals based on the likelihood ratio can be found in Royall (1997), while Bandyopadhyay et al. (2016) discuss ΔSIC evidential intervals. As with ΔAIC, there are some guidelines (suggestions) on what constitutes weak evidence or strong evidence for one model over another based on the value of ΔSIC. Raftery (1995) suggested that a ΔSIC (i.e., ΔBIC) values less than 2, 2 to 6, 6 to 10, and greater than 10 constitute weak, positive, strong, and very strong evidence, respectively. Such verbal partitioning of any information criterion is often desirable for interpretation, but rarely justified.

Box 3 provides a more intuitive probabilistic approach to selecting a value. From our more detailed example in Box 3 using binomial probability model, it can be shown that at five consecutive heads, the probability of this occurring by chance is ∼0.03 with a ΔIC∼7. Building an uncertainty bound around a parameter value requires choosing a ΔSIC value, we use seven as our threshold for intervals, ΔSIC(7).

**BOX 3. Intuitions about evidence.**

Fisherian significance tests (think *p*-values) and Neyman-Pearson hypothesis test (think α levels) rely on critical values. The confusion and convolution of these two statistical approaches have led applied scientists to misinterpretations of the strength of evidence against the null hypothesis. As Hubbard and Bayarri (2003) so state it, “This mass confusion, in turn, has rendered applications of classical statistical testing all but meaningless among applied researchers.”

Multi-model inference using Information Criteria (IC) (e.g., AIC, SIC) have a continuous measure of evidence found in the difference (i.e., ΔAIC, ΔSIC) in values between the best model (hypothesis) and the reference model. However, communicating this strength of evidence has resulted in vagueness emerging from linguistic uncertainty (Elith et al., 2002). This is to say, applied scientists have created guidelines to discuss the strength of evidence. Maybe the most popular recommendation was provided by Burnham and Anderson (2002) for ΔAIC (AIC_i_ – AIC_j_), where 0 > ΔAIC > 2, 4 > ΔAIC > 7, ΔAIC > 10, represent “substantial,” “considerably less,” and “essentially none” levels of evidence to support for retaining model *i* in the model set along with the best model *j*. Never minding the absence of what a value of 3 might indicate, some scientists have suggested different discretization of intervals (i.e., Burnham et al., 2011) adding to the apparent vagueness of what constitutes evidence on a continuous scale rather than a discrete critical test provided by *p*-values (Murtaugh, 2014).

To a certain extent that different scientists recognize different ΔIC levels as strong evidence represents differences in attitude about science as a whole and their specific research problem. This variation is no different from one scientist choosing a critical value of 0.05 for a hypothesis test and another scientist choosing 0.01. The clearest exposition for developing an intuition for evidence on a continuous scale (Box 2, D2) for an evidence function is in Royall (1997), which we recast here in terms of coin tosses.

Imagine that you are gambling with someone on their flipping of a coin and wonder if you are being cheated with a double-headed coin, or if the coin is fair. After the first coin toss results in a head you are not worried, yes there is a small amount of evidence for a double-headed coin, but it is just a single coin toss. Two heads in a row still happens frequently. With three heads in a row your suspicions are peaked. By four heads in a row you are having serious doubts. Five heads in a row pretty well convinces you that you are being cheated. And, after seeing eight heads in a row you are reaching for the derringer in your boot.

We can augment this example with calculations of the *p*-value of so many heads under the null model of a fair coin. Fisherian significance testing is generally the first inferential tool that we are taught so many of us will have developed intuitions on *p-*values. In the calculation of the *p*-values, the null model is the fair coin model. Evidence is often measured as a likelihood ratio. The table shows the ratio of the likelihood of the double headed coin model given the data to the likelihood of the fair coin model given the same data. We can scaffold these intuitions into greater understanding of the evidence contained in differences in information criteria, ΔIC = (2^∗^Log(Likelihood ratio)). Selecting a specific IC, such as AIC or SIC, would introduce a penalty term for the number of parameters and amount of data (Eq. 2).

**Table:** 

**Consecutive heads**	***p*-value**	**Likelihood ratio**	**ΔIC**	**Evidence intuition**
1	0.5	2	1.39	Very weak
2	0.25	4	2.77	Weak
3	0.125	8	4.16	Marginal
4	0.063	16	5.55	Moderate
5	0.031	32	6.93	Strong
6	0.016	64	8.32	Very strong
7	0.008	128	9.70	Extremely strong
8	0.004	256	11.09	Overwhelming

Expectedly, there is a common trend between the *p*-value and ΔIC. As the evidence grows for a two-headed coin, the *p*-value gets smaller, while the ΔIC value increases. In Fisherian *p*-value testing, we would have selected a threshold for the observed data (say 0.05) that beyond which we would reject the null model (hypothesis) in favor of the alternative. Interpretation of *p*-values is generally not condoned as a strength of evidence. With the ΔIC, we have a gradient from which to draw our inferences.

We see at a *p*-value of 0.031, the ΔIC is 6.93. For our study, we selected ΔSIC(7) for our intervals – meaning models and values of the slope parameter within this bound should be retained for further consideration with more data. Models and values of the slope parameters outside this bound have strong evidence against those models giving rise to the observed data (relative to the best model) and can therefore be subsequently dismissed.

**FIGURE 2:**
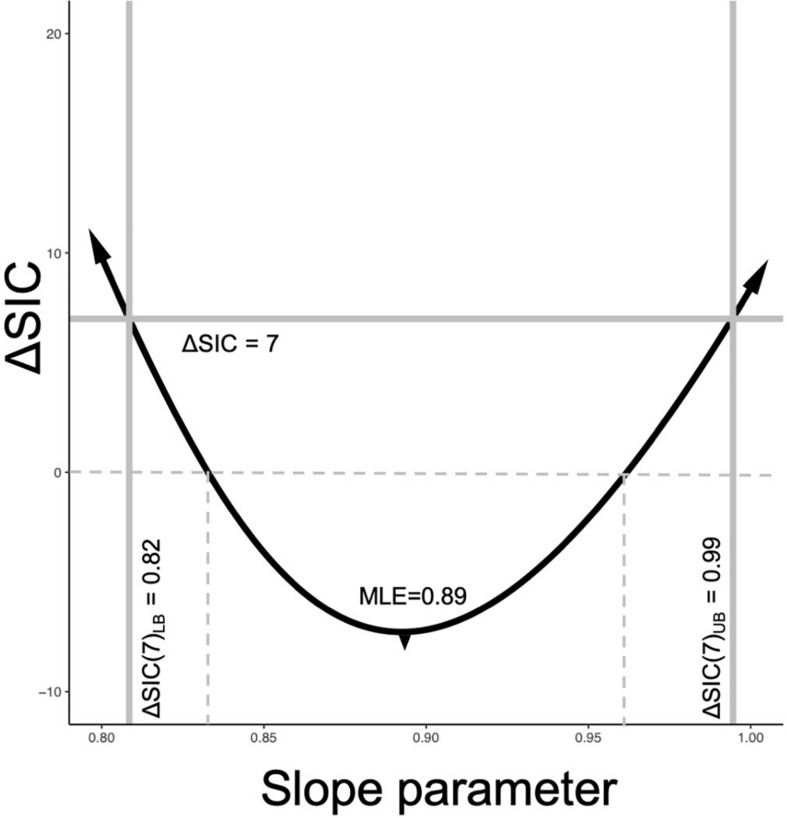
SIC interval formulation. The black line is the ΔSIC as a function of the slope parameter space. The reference model is always the model with the estimated slope parameter. When ΔSIC = 7 (solid gray horizontal line intersects the ΔSIC), this defines the lower ΔSIC(7)_LB_ and upper ΔSIC(7)_UB_ of the information criterion interval. Values of the ΔSIC near the MLE can be negative values due to the penalization term (Eq. 2). This example is drawn from the best fit model of our study with an MLE for the slope parameter of $\hat{\beta }=0.89$ with ΔSIC(7) = (0.82, 0.99). When the ΔSIC is negative, that is below the dashed line, the fixed slope models are favored, but weakly. When the ΔSIC is positive but less than 7, fitted slope model is favored, but weakly.

A ΔSIC interval for the metabolic scaling relationship (slope parameter) can be built for each trial or for the best selected model by calculating ΔSIC across the parameter space of the slope parameter. The ΔSIC is the difference of the SIC of the best model and the SIC of the same model with a fixed value of the slope parameter. The upper and lower bound of the ΔSIC interval occurs when ΔSIC = 7. Figure 2 visually captures the process, where the parameter space of the slope parameter is on the x-axis and the ΔSIC is a function of this slope parameter. Expectedly, ΔSIC values greater than 7 would result in broader intervals. If we consider ΔSIC(7) as strong evidence, then the bound can be interpreted as *there is strong evidence that values of the scaling relationship outside of this range do not give rise to the observed data.* For purpose of our study, we provide ΔSIC(7) intervals for each trial and for the best model. In practice, models with parameter values falling within the ΔSIC interval are cases where, given the data in hand, the scientist cannot strongly differentiate between the models within the bound, and all of these models should be retained and further scrutinized with additional data (Box 2, D6).

## Results

**FIGURE 3:**
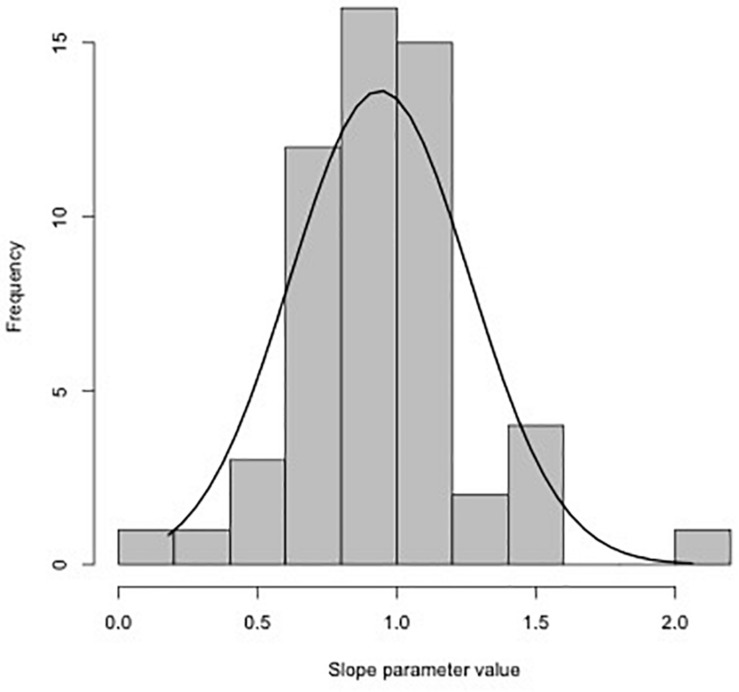
Distribution of slopes estimated in Table 1 for all 55 trials. Mean of the distribution is 0.94 (SE 0.04).

Using the slopes estimated for each trial (Table 1), the distribution of values with fitted normal curve is shown in Figure 3. The mean slope parameter value is 0.94 (SE 0.04), which is unexpectedly different than the 0.79 slope estimated from the synthesis provided by Clarke and Johnston (1999). One explanation for this difference is because many of the studies used in our analysis were initially conducted to test the SMR of similar body sized fish at different temperatures. As indicated by trial 28 (Table 1), small sample size (*n* = 8) can result in biologically unrealistic estimates $\left(\hat{\beta }=-0.21\right)$.

The best model selected using ΔSIC came from model suite 3 with a random intercept and random slope, but with a common slope parameter of $\hat{\beta }=0.89$ (SE 0.021). However, a common slope and random intercept model had a ΔSIC = 1.5, and is thus not strongly distinguishable from the best model. The correlation of random slope with random intercept was −0.86, indicating that as the intercept increases in value, the slope decreases in value. This correlation is likely due to noise.

The value of universal slope is consistent (0.87–0.89) across all model suites and there is strong evidence (ΔSIC > 7) against fixed mechanistic based values of the metabolic scaling rate of 0.67, 0.75, and 1 across all modeling suites. Figure 2, along with being a conceptualization of an ΔSIC(7) interval, is generated under the best model and the interval spans 0.81 to 0.99.

**FIGURE 4:**
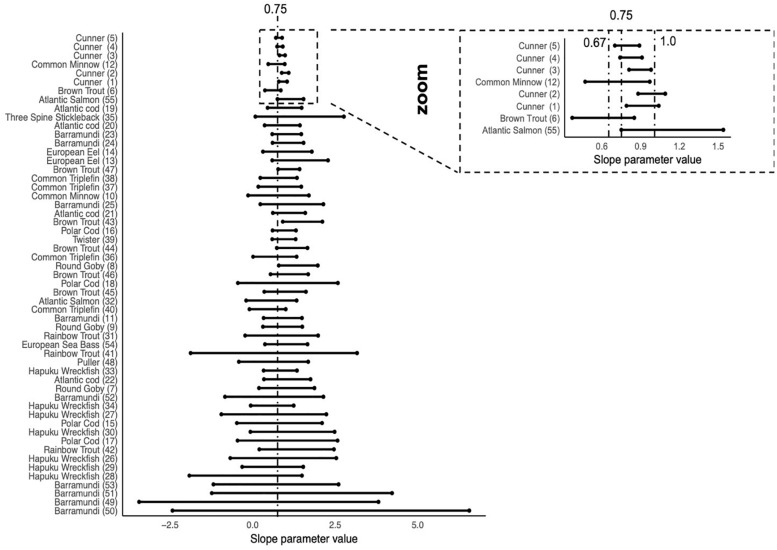
ΔSIC(7) intervals for all trials ordered by n^∗^VAR (Log(weight)). Trials with small n^∗^VAR(Log(weight)) are expected to have wide intervals because the lack coverage of fish mass or have small samples sizes. As studies have larger n^∗^VAR(Log(weight)), the ΔSIC(7) intervals become smaller and have the ability to exclude hypotheses of the slope, *β* = 0.67, 0.75, and 1. With the exception of the Cunner(3) trial, all other trials capture at least one of the hypotheses, the most common being *β* = 0.75, the dashed line in the figure. The zoom inset shows trials with relatively narrow ΔSIC(7) and dashed lines at *β* = 0.67, 0.75, and 1.0.

Figure 4 shows the ΔSIC(7) interval for each trial ordered by n^∗^VAR(ln(weight)), from smallest values at the bottom to larger values at the top. This ordering is a regression experimental design component where few data points and/or small ranges in body mass result in small values indicating the lower precision of the slope parameter estimate. With exception of Cunner (Trial 3) where the ΔSIC(7) interval spans 0.81 to 0.98, all other trials span at least one of the mechanistic hypotheses of 0.67, 0.75, or 1.

As outlined in the data section, all observations included in this study were collected under conditions to ensure data quality. However, not all studies were designed to estimate metabolic scaling relationship (a slope parameter) and some had few data points and/or did not cover a large breadth of fish body masses. The trials of Cunner, however, were designed for testing the metabolic scaling relationship and could potentially drive the overall value observed by the best model. As such, we conducted an additional analysis after removing the Cunner data and found the same estimate of the metabolic scaling relationship. See Box 4 for more details. The metabolic scaling relationship of $\hat{\beta }=0.87-0.89$ for fish has very little uncertainty, is robust across models, and emerges when any trial or species is dropped from the analysis.

**BOX 4. Is it just cunner?**

The Cunner study (Norin and Gamperl, 2018; *n* = 66 per trial for five trials) and the Common Minnow (McLean et al., 2018; *n* = 122 for one trial) both have large sample sizes compared to the other studies and were intentionally designed to estimate the metabolic scaling. Consequently, when we look at the span of ΔSIC(7) interval estimated for each trial as a function of the regression experimental design measure *n* ^∗^ the variance of Log(weight) (Figure 4), we see the Cunner and the Common minnow studies have distinctly smaller ΔSIC(7) intervals. This raises the question, would our conclusion about the value of intraspecific scaling coefficient if the cunner study or the Common Minnow study were not included in our analysis.

We estimated the slope parameter under the best fit model and then calculated the resulting ΔSIC(7) interval by systematically withholding data by trial and then by species. For trials (Figure Box 4.1), they are ordered by value of *n* ^∗^ the variance of Log(weight) from largest to smallest. For species (Figure Box 4.2), the ordering is alphabetical.

**FIGURE BOX 4.1:**
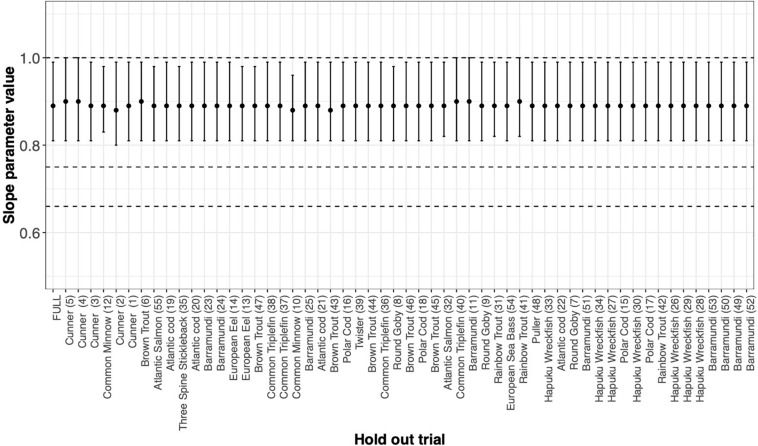
MLE of the slope parameter and ΔSIC(7) interval estimated by systematically withholding each species. FULL is the MLE and interval with all data considered. Absence of any one data set does not drive our conclusion. However, absence of Barramundi, Common Triplefin, Cunner, Hapuku Wreckfish, or Rainbow Trout would suggest keeping the mechanistic hypothesis of metabolic scaling at 1 in the suite of models to be considered further.

**FIGURE BOX 4.2:**
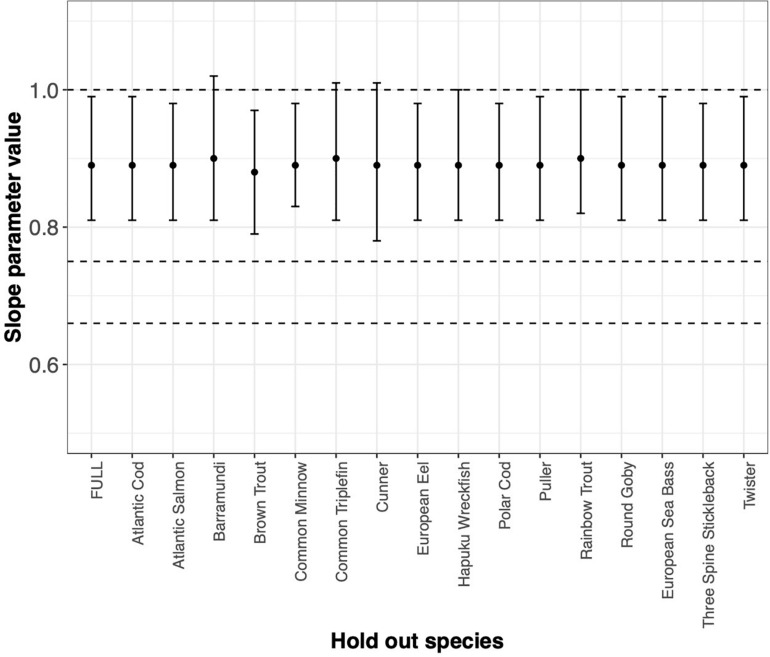
MLE of the slope parameter and 1SIC(7) interval estimated by systematically withholding each species. FULL is the MLE and interval with all data considered. Absence of any one data set does not drive our conclusion. However, absence of Barramundi, Common Triplefin, Cunner, Hapuku Wreckfish, or Rainbow Trout would suggest keeping the mechanistic hypothesis of metabolic scaling at 1 in the suite of models to be considered further.

As expected, Cunner trials and the Common Minnow trial indeed do influence the MLE and the ΔSIC(7) intervals (Figure Box 4.1), but not so much as to capture the mechanistic hypotheses of 0.75 and 0.67 (dashed lines). However, the full model inference that the mechanistic hypothesis of metabolic scaling = 1 can be excluded from further consideration is sensitive to inclusion of some trials and species (Figure Box 4.1 and Figure Box 4.2). In all trials, the value of $\hat{\beta }=0.89$ is captured. Other trials with smaller values of *n* ^∗^ the variance of Log(weight) have virtually no influence on the either the point estimate or the uncertainty measure.

The story is similar if we aggregate trials by species (Figure Box 4.2) and then systematically withhold all data from a species. Notably, withholding species data generally broadens the ΔSIC(7) interval with slight variation in the MLE that ranges from 0.89 to 0.9. Yet withholding a species from the analysis does not change the conclusion of the statistical inference that the slope of the metabolic scaling relationship is not 0.75 or 0.67. However, absence of Barramundi, Common Triplefin, Cunner, Hapuku Wreckfish, or Rainbow Trout results in a wider ΔSIC(7) interval that just captures the metabolic scaling of 1, and would, in the absence of any of these species, motivate further consideration of this mechanistic hypothesis.

While some of the trials were designed to test the metabolic scaling relationship, they do not unduly drive the conclusion. But maybe more importantly, the effect of many studies that are less suited to individually test the relationship (Table 1), together can provide meaningful insights into the metabolic scaling relationship.

## Discussion

The evidence function (ΔSIC) approach we implemented here has led to selecting a best model; a mixed effect model with random slope and random intercept by species and an estimated correlation between random effects (Table 2, Model 9). However, we cannot dismiss the possibility that the model structure may only have a random species intercepts and common slope as witnessed by this alternative model having a ΔSIC = 1.5 (Table 2, Model 1). Models across all suites that represent mechanistic hypotheses of a scaling relationship of 0.67, 0.75, and 1 are dismissed with *very strong* evidence, ΔSIC > 8.4 (Table 2, Box 2). As such, our inference is that surface area limitations (*β* = 0.67), distribution network limitations (*β* = 0.75), and low cost demands on maintenance and routine activity (*β* = 1) are not exclusively driving the metabolic scaling relationship in fish.

**TABLE 2:** Application of evidence functions using the Schwarz Information Criterion (SIC).

**Model**	**Description**	***k***	**Log(L)**	**SIC**	**Δ SIC**
**Model Suite 1: Random Intercept models.**					

**Fixed effect: Weight, Temp; Random effect: Species; Nested effect: Trial**					
1	*β* = Free^∗^	6	73.9	–104.1	1.5
2	*β* = 0.67	5	–15.5	67.4	173
3	*β* = 0.75	5	42.1	–47.7	57.9
4	*β* = 1	5	35.7	–35	70.6

**Model Suite 2: Estimated species intercept models**					

**Fixed effect: Weight, Temp and Species, Nested effect: Trial**					
5	*β* = Free^∗^	20	–69.8	–69.8	35.8
6	*β* = 0.67	19	96.8	96.8	202.4
7	*β* = 0.75	19	–16.3	–16.3	89.3
8	*β* = 1	19	–0.4	–0.4	105.2

**Model Suite 3: Random intercepts with random slopes**					

**Fixed effect: Weight and Temp; Random effect: Species and Slope; Nested effect: Trial**					
9	*β* = Random, Free^∗^	8	81.9	–105.6	0
10	*β* = Random, 0.67	7	50.1	–49.2	56.4
11	*β* = Random, 0.75	7	64.6	–78.1	27.5
12	*β* = Random, 1	7	74.1	–97.2	8.4

**Model Suite 4: Estimated species intercept models with random slopes**					

**Fixed effect: Weight, Temp and Species; Random effect: Slope; Nested effect: Trial**					
13	*β* = Random, Free^∗^	21	107.8	–62.6	43
14	*β* = Random, 0.67	20	45.2	55.3	160.9
15	*β* = Random, 0.75	20	78.6	–11.5	94.1
16	*β* = Random, 1	20	85.7	–25.6	80

*^∗^R output with parameter values found in the Supplementary Material.*

However, the evidence for a $\hat{\beta }=0.87$ to 0.89 universal scaling relationship is strong and presumably robust as indicated by similarity of the MLE for this parameter across all modeling suites and narrow bound of the ΔSIC(7) interval (Figure 2). Both fixed values are more than five standard deviations from the estimated common slope, and thus the chances are less than 1 in 1,000,000 that the common slope would have a *β* as small as 0.75 or as great as 1. If the data do come from the random slopes model, then it would be an extraordinary event for any species to have a *β* as low as 0.75, but perhaps as much as 6% of species might have a *β* as great 1. Accordingly, both DEB and MLB hypotheses warrant further consideration to determine the mechanism of metabolic scaling in fishes.

In many ways, the evidentialist approach is not that different from what is being applied in the multi-model literature, albeit with the meaningful caveat that an evidence function (Box 2) is being applied. The SIC is well studied, familiar to many, and also extractable from all the analyses we conducted in the R programing language. As such, the ΔSIC is readily accessible to scientists wishing to implement an evidentialist approach. While additional coding is required to produce ΔSIC intervals, this effort takes only elementary coding to automate. It must be noted, that the SIC for large sample sizes makes it difficult for new parameters to enter the model. In this analysis, our primary conclusion is that a model with β estimated as an extra free parameter is better than any of the models with β specified at any of the values of 0.67, 0.75, or 1.0. Thus the use of the SIC as a criterion as opposed to the AIC makes our conclusions conservative.

The other major contribution of the evidentialist approach underscored in this is the imperative to combine data sets such that evidence does not come from a single critical test, but rather from the accumulation of trials and critical tests (See D6 of Box 2). Here we combined 55 trials across 16 species comprising 1456 observations. While this would normally form the basis of meta-analysis, this breadth of diverse data is desirable by allowing for a random effect of species to make our inferences across the population of fish species. If we look at each trial individually, we see that all but one trial (Cunner 4), captures one of the mechanistic hypotheses of 0.67, 0.75, or 1. In contrast when we look at the aggregate, none of these hypotheses are supported (Box 4).

Both the quantity and quality of metabolic rate data included in the metadata are important and can shape the conclusions of the study. Several extensive metadata analyses include mean metabolic rate values from close to a 100 or more species (e.g., Clarke and Johnston, 1999; White and Seymour, 2003; Glazier, 2005; Killen et al., 2010); however, the methods and quality of the data is not always rigorously considered. Metabolic rate is one of the most commonly investigated whole animal physiological performance metrics (Nelson, 2016), but different methods are more or less time and resource-intensive and can over-estimate SMR (Chabot et al., 2016). Furthermore, it is logistically challenging to obtain robust SMR measurements on many fish species, for example, large-bodied open ocean pelagic species or deep-sea fishes. Our study is unique because we only included standard metabolic rate data following specific and stringent criteria with each data point representing *individual* standard metabolic rate instead of reported species mean values. Future work could address how our (and others) conclusions change if the quality control criteria are relaxed.

There are many covariates that may be important predictors for species-specific scaling slopes and intercepts. While we tried to capture fishes across a broad latitudinal range with varying life histories, we did not examine life history factors such as species ecological activity (athletic vs. sedentary; Killen et al., 2010), growth rate, reproductive investment or strategy (e.g., fecundity), maximum body size, maximum age, or even environmental factors such as habitat (e.g., benthic vs. pelagic; freshwater vs. marine; Killen et al., 2010), or latitude (e.g., tropic vs. temperate vs. polar). Furthermore, temperature governs metabolism in ectotherms such as fish. Given this, all our models included temperature as an independent significant predictor of metabolic rates in fish (ΔSIC = 8.1 for best model compared to best model without temperature; Box 5). Recently, Lindmark et al. (2018) presented temperature-dependent intraspecific metabolic allometry, where MR increased with temperature to a lesser extent in larger fish. Furthermore, these effects scale to higher levels of organization, including from populations (population response-models), to ecosystems (MTE; Brown et al., 2004). We evaluated temperature effects and an interaction with log(weight) (See Box 5) with a ΔSIC = 7.2 compared to the best model. We can dismiss further consideration of an interaction of temperature with weight under the model suites evaluated. However, these temperature-size dependent effects on MR are mixed across and within species, and require more research and metabolic scaling data from species in polar and tropical environments.

**BOX 5. Changes in SMR due to temperature and body mass.**

Temperature has been thought to play a critical role regulating individual metabolic rate in fishes (Fry, 1947), where metabolic rates typically increase as temperature increases. As a consequence, all of the models we have considered so far have included a temperature effect. We can evaluate the effect of temperature more fully by considering six modification of models in suites 1 and 3 (Table 2). The first model (M17) is a random intercept model without inclusion of the temperature variable. The second model (M1) includes temperature (Table 2, Suite 1, Model 1), and the third model (M18) adds an interaction term of temperature with log(weight). These are all fixed slope models.

Including a log(weight) by temperature interaction is equivalent to saying that scaling of log(SMR) with log(weight) is itself a linear function of temperature. This is how we express it in the table below. The derivation of the standard error is discussed in the Supplementary Material.

The second group of models are built upon the random slopes model (Table 2, Suite 3, Model 9). The first model (M19) is absent temperature, the second model (M9) is the same as Table 2, Model 9 with an intercept defined by the temperature, and the third model (M20) has an interaction of temperature with log(weight). Using maximum likelihood fitting and extracting the SIC values, we can apply the same evidence function approach to evaluate the influence of temperature on intraspecific metabolic scaling. Model output is provided in the Supplementary Material.

Consistent with our previous model selection effort, M9 (Table 2), which includes temperature with a metabolic scaling coefficient (0.89), has the lowest SIC score. Models M17 and M19 without temperature include have ΔSIC > 7, which indicates temperature is a significant factor as the literature suggests. As observed previously, there is moderate evidence for M9 over M1, but not so much as to discourage future studies from considering a constant slopes model. Both M18 and M20 with interactions between temperature and log(weight) have ΔSIC > 7. Under the best model (M9), the expected metabolic scalings at 0°C, 15°C, and 30°C are 0.89, 0.9, and 0.91, respectively.

The conclusion from our focused study of temperature is that temperature is a critical factor to consider in modeling fish metabolic rate as there is *strong* evidence (Box 3) for including temperature in the intercept of the scaling relationship. Future work on evaluating the effect of temperature should expand the coverage of the temperature range with more polar and tropical fish species. Additional data at the endpoints of the temperature range will improve inferences about the scaling relationship and the evidence for, or against, a log(weight) by temperature interaction.

**TABLE BOX 5.1:** Model selection using ΔSIC along with parameter estimates of for the metabolic scaling relationship. For models M18 and M20, the parameter estimate and standard error are a function of temperature.

**Model**	**SIC**	**ΔSIC**	**$\hat{\beta }$**	**SE($\hat{\beta }$)**
M17	–80.6	25	0.87	0.015
M1	–104.1	1.5	0.87	0.015
M18	–97.5	7.6	0.83 + 0.00257 (temp)	$\sqrt{0.0023+\left(8.59\times 10^{-6}\right)\times {\text{temp}}^{2}+2\times -0.00013\times \text{temp}}$
M19	–86.1	8.1	0.91	0.025
M9	–105.6	0	0.89	0.021
M20	–98.4	7.2	0.87 + 0.00106 (temp)	$\sqrt{0.0033+\left(1.07\times 10^{-5}\right)\times {\text{temp}}^{2}+2\times -0.00017\times \text{temp}}$
				

Norin and Gamperl (2018) provided a compelling study to measure allometric scaling for Cunner. It adhered to all the characteristics of a robust and well-designed study (White and Kearney, 2014) to estimate the scaling relationship, with ample breadth of fish mass, 68 observations per trial, and five trials (Table 1). What makes this study notable is their conclusion that no universal scaling relationship exists. We offer a few explanations for this apparent contradiction. Our inference is broadly applicable to fish, while theirs is limited to Cunner. Put simply, we are measuring evidence at a different inferential level for a universal scaling constant. If we look at the values of the SIC(7) intervals for all Cunner trials (Figure 4) they appear to be very similar. The intervals are {0.79, 1.04}, {0.88, 1.09}, {0.81, 0.98}, {0.74, 0.91}, and {0.7, 0.89}, and all SIC(7) intervals capture the values 0.88 and 0.89. Clearly our estimate of $\hat{\beta }$ = 0.89 from the best model with a random slope should be considered as a possible universal scaling for Cunner as well as other fish. As such, our results are consistent with Norin and Gamperl (2018), and their insightful suggestions about the need to consider species-specific scaling relationships when building fish population dynamic models that apply metabolic scaling exponents, should be heeded.

Scaling relationships are at times considered key tools for predicting the effects of global change on fisheries (e.g., Cheung et al., 2008), or as tools to estimate how abundant fish might be in the absence of fishing (e.g., Jennings and Blanchard, 2004). Therefore, variation in the scaling relationship between body size and metabolism have clear implications for how we predict fish populations will respond to changes in the environment or changes in body size distributions. As we move forward and seek to predict the consequences of changes in fish populations, the assumption of a universal scaling exponent, while attractive and generalizable may either under or overestimate a species sensitivity to changes in the environment. Given the evidence for species-specific variation in scaling relationships provided in our study, stock assessments seeking to integrate scaling relationships into forecasts may therefore benefit from species-specific values. While theoretical underpinnings have motivated application of a scaling relationship of *β* = 0.75, our data show that fisheries models that blindly adopt this parameter may be ultimately misleading.

We had some concern that the species distribution would be non-normally distributed, but there was no evidence from our analysis of this concern. However, those models may be useful for assessing the importance of species phylogenetics to metabolic scaling. The variance for the random species intercept model was 0.19 with a residual of 0.047. Similarly, from the random slopes model, the variance for the random intercept was 0.24, the random slope was 0.005, and the residual variance was 0.044 (see Supplementary Material for model outputs). Both measurement error in SMR and real inter-species variability contribute to the variability in $\hat{\beta }$. Variance components are notoriously difficult to tease apart, that is they are only weakly estimable (Ponciano et al., 2012). An estimate of the magnitude of measurement error in SMR would contribute greatly to the ability of further studies to accurately estimate the inter-specific variability in $\hat{\beta }$.

This study does not address the question of inter-specific metabolic scaling. This would entail a study of scaling of intra-specific intercepts with mean species body size. As we do not have accurate estimates of mean body size for these species, we cannot yet address this issue. Future work could use the random affects models or the estimated species intercepts models (model suites 2 and 4, Table 2) to evaluate if species relatedness and/or taxonomy are significant factors explaining species random effects variability.

Many of the studies used in this analysis were not designed to test the metabolic relationship, which is evident from the standard errors of the regression coefficients for individual trials (Table 1). However, under our data criteria, these studies had precise measurement of SMR, body mass, and temperature. The inclusion of these trials added unique species to support the evaluation of a species random effect, which ultimately allows us to make inferences from this model across fish species. Given that some of these trials are ill-suited in themselves to critically test the metabolic relationship, due to low sample size or narrow range of body masses, this may be contributing to selection of the random slope model. Future studies that implement an evidentialist approach with additional data sets collected using appropriate experimental designs to uncover the allometric scaling relationship will likely reconcile if species requires a random slope.

Simulations to understand data requirements for robust analysis of interspecific metabolic scaling relationships suggest that the data should include 100–150 species spanning 3–4 orders of magnitude range in body size (White and Kearney, 2014). One approach to finding or estimating a universal intraspecific scaling constant is to take the average from the distribution of estimated slopes from each trial (e.g., Figure 3 in the current study, 0.916, SE 0.04). This approach, while easy to implement by combing the literature, assumes that all data are created equal, but we know that each estimated slope, $\hat{\beta }$ comes with error, and some of the studies we included had relatively large standard errors (Table 1). Our data with fewer total species than most meta-analysis, but using individual data instead of species or trial means, proved to be sufficient to address the question concerning the universality of scaling relationship between fish body mass and metabolic rate.

The evidentialist approach is useful in addressing long-standing scientific debates (such as universal scaling relationships of metabolism), consistent with the practice of applied scientists, and relatively easy to implement using existing evidence functions and programing packages. It provides path forward for dismissing models (hypotheses) with little to no support, identifying and retaining hypotheses needing further evaluation, and provides a philosophy that emphasizes accumulation of evidence, through additional data and confronting that data with more complex models of how the nature works. We look forward to further refinement of the approach not only through philosophical insights and mathematical rigor, but through application of the approach to long-standing, pressing ecological and environmental science problems.

## Data Availability

The data sets analyzed for this study, with exception of the measurement error data for lobster, are peer-reviewed and published (see Table 1 for citations). Data sets are available from the originating author(s). The lobster data are available in the Supplementary Material.

## Author Contributions

All authors contributed to the writing of the manuscript. MT, CJ, EE, and KK conceptualized the project. CJ and MT conducted the analyses. EE and KK created the database. SC and KK collected the lobster data.

## Conflict of Interest Statement

The authors declare that the research was conducted in the absence of any commercial or financial relationships that could be construed as a potential conflict of interest.
