# Supplementary material for: Strong Evidence for an Intraspecific Metabolic Scaling Coefficient Near 0.89 in Fish
Source: Front Physiol. 2019 Sep 20;10:1166. doi: 10.3389/fphys.2019.01166 (PMC6763608; doi:10.3389/fphys.2019.01166)
Supplement: Supplementary file 1 [file Data_Sheet_1.pdf]

Supplementary material

Supplementary material 1: By species regressions

Atlantic Cod

(Gadus morhua)

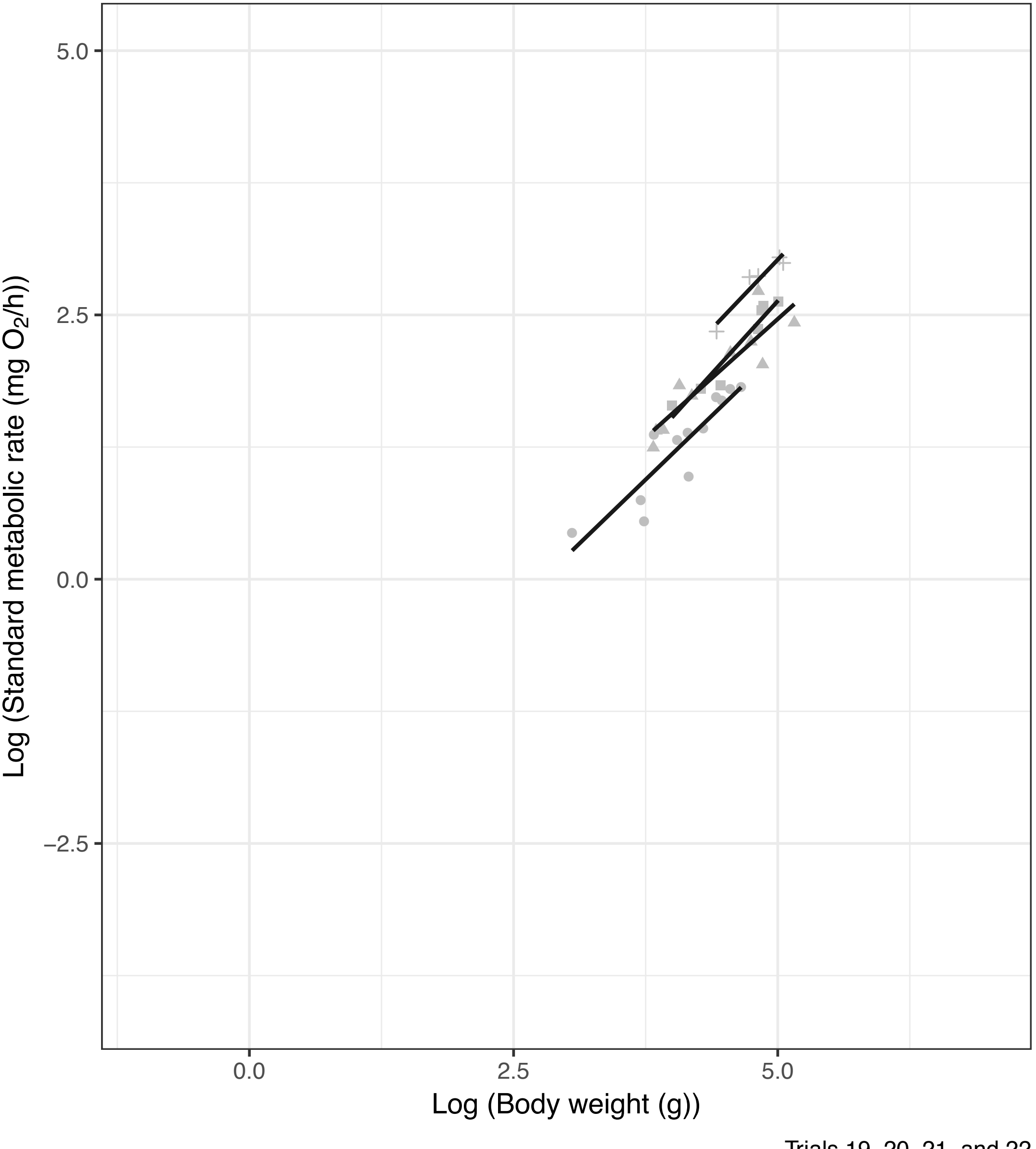

Trials 19, 20, 21, and 22

Atlantic Salmon

(Salmo salar)

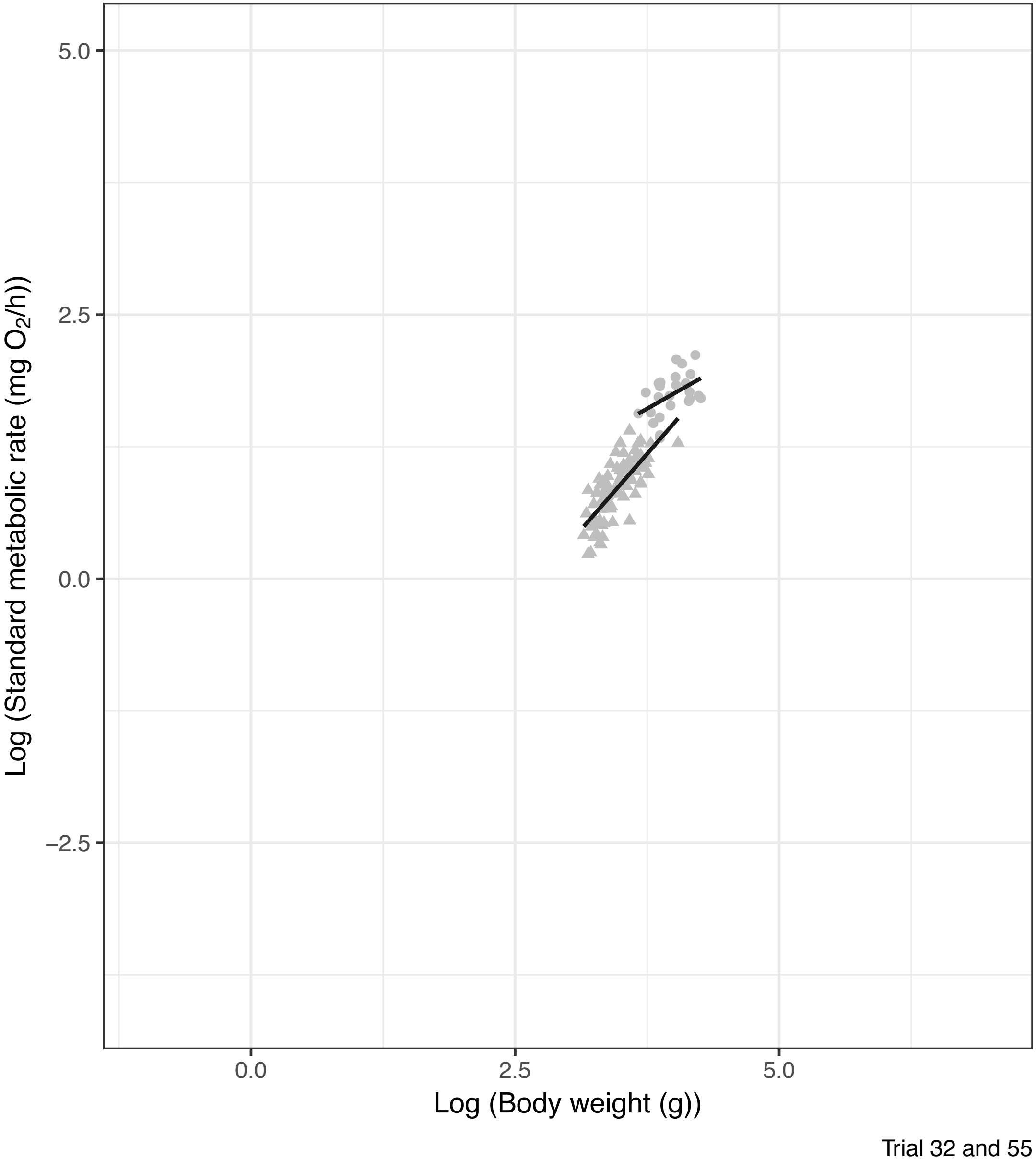

Trial 32 and 55

Barramundi

(Lates calcarifer)

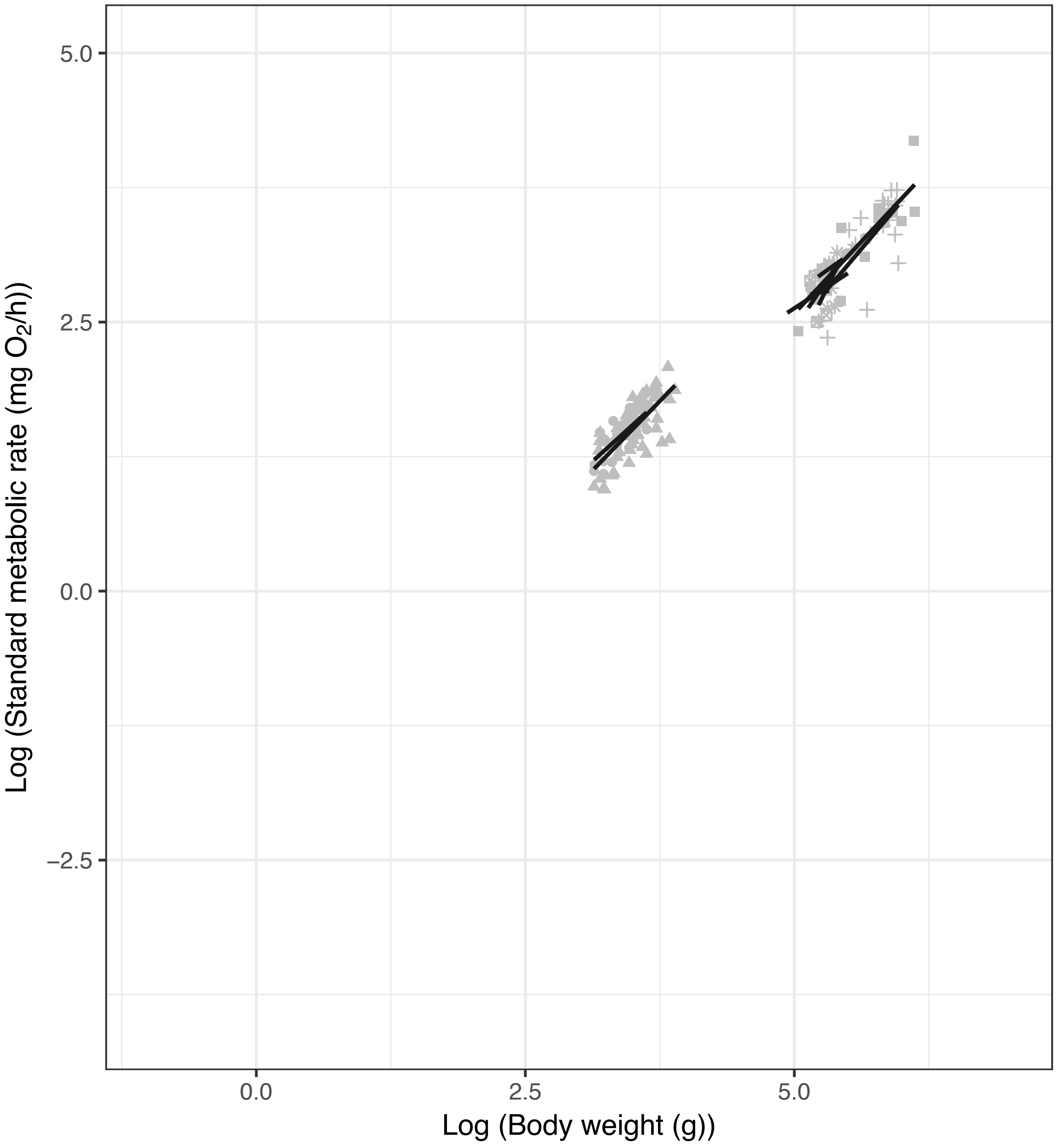

Trials 11, 23, 24, 25, 49, 50, 51, 52, and 53

Brown Trout

(Salmo trutta)

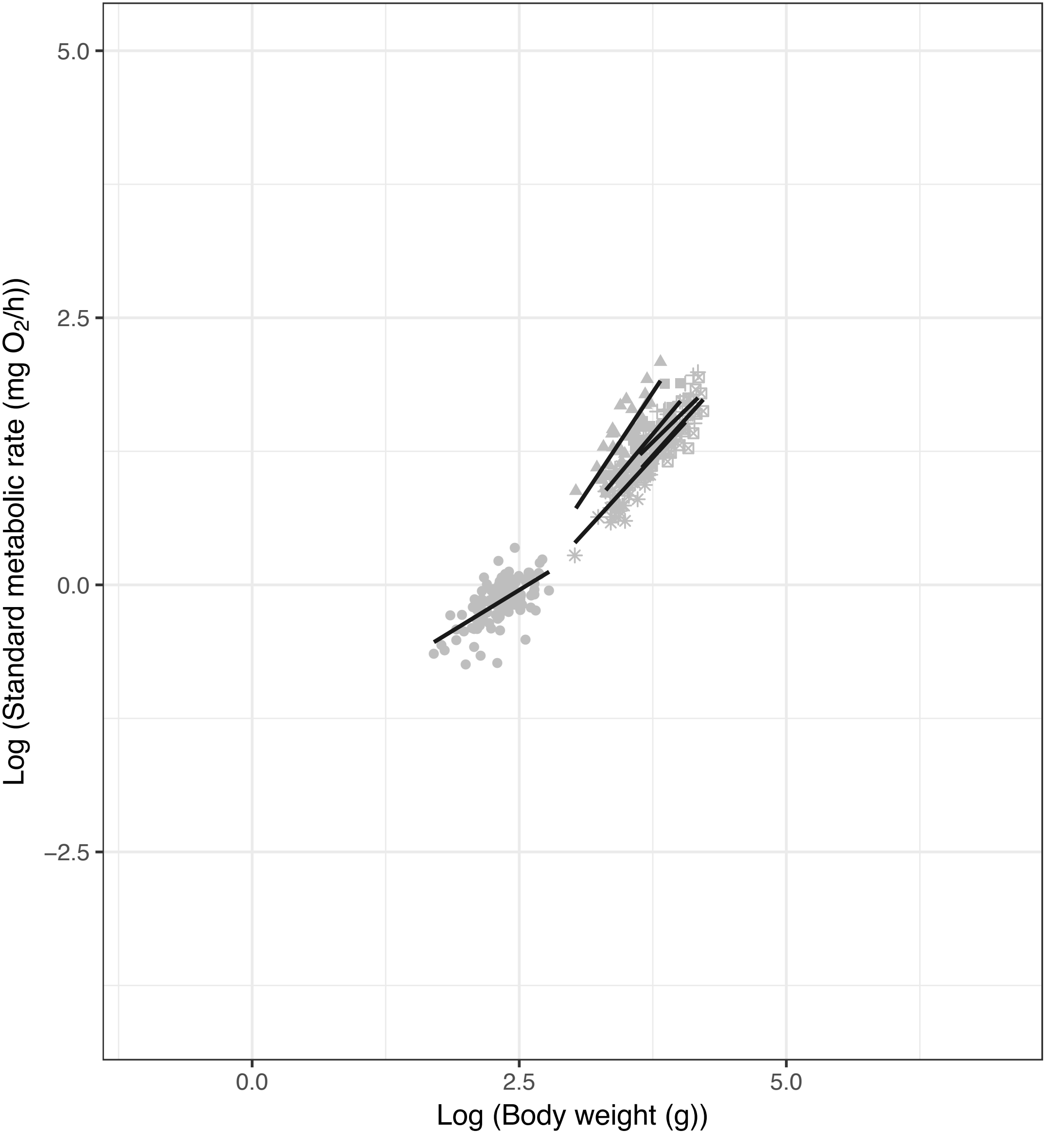

Trials 6, 43, 44, 45, 46, and 47

Common Minnow

(Phoxinus phoxinus)

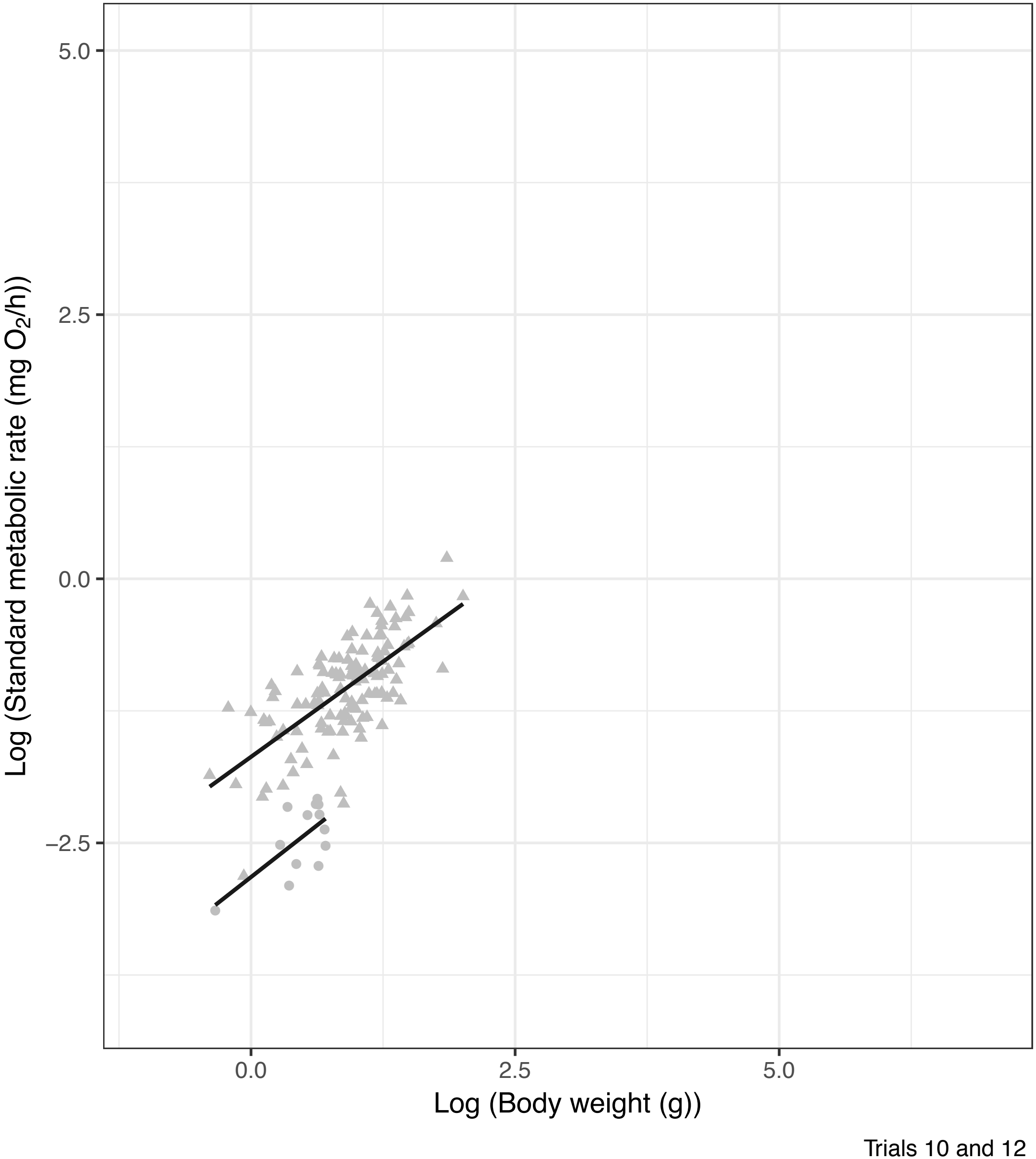

Trials 10 and 12

Common Triplefin

(Forsterygion lapillum)

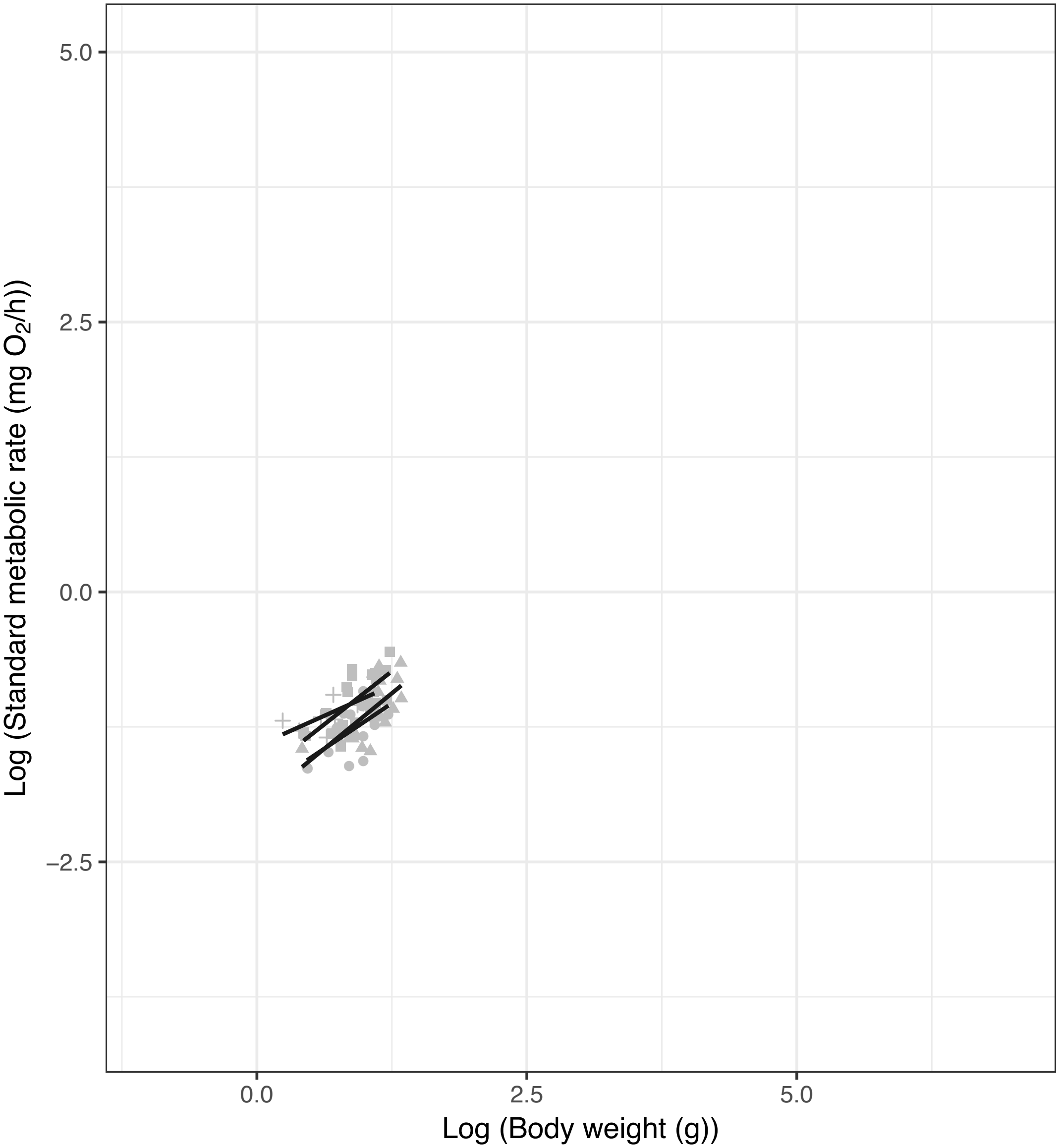

Trials 36, 37, 38, and 40

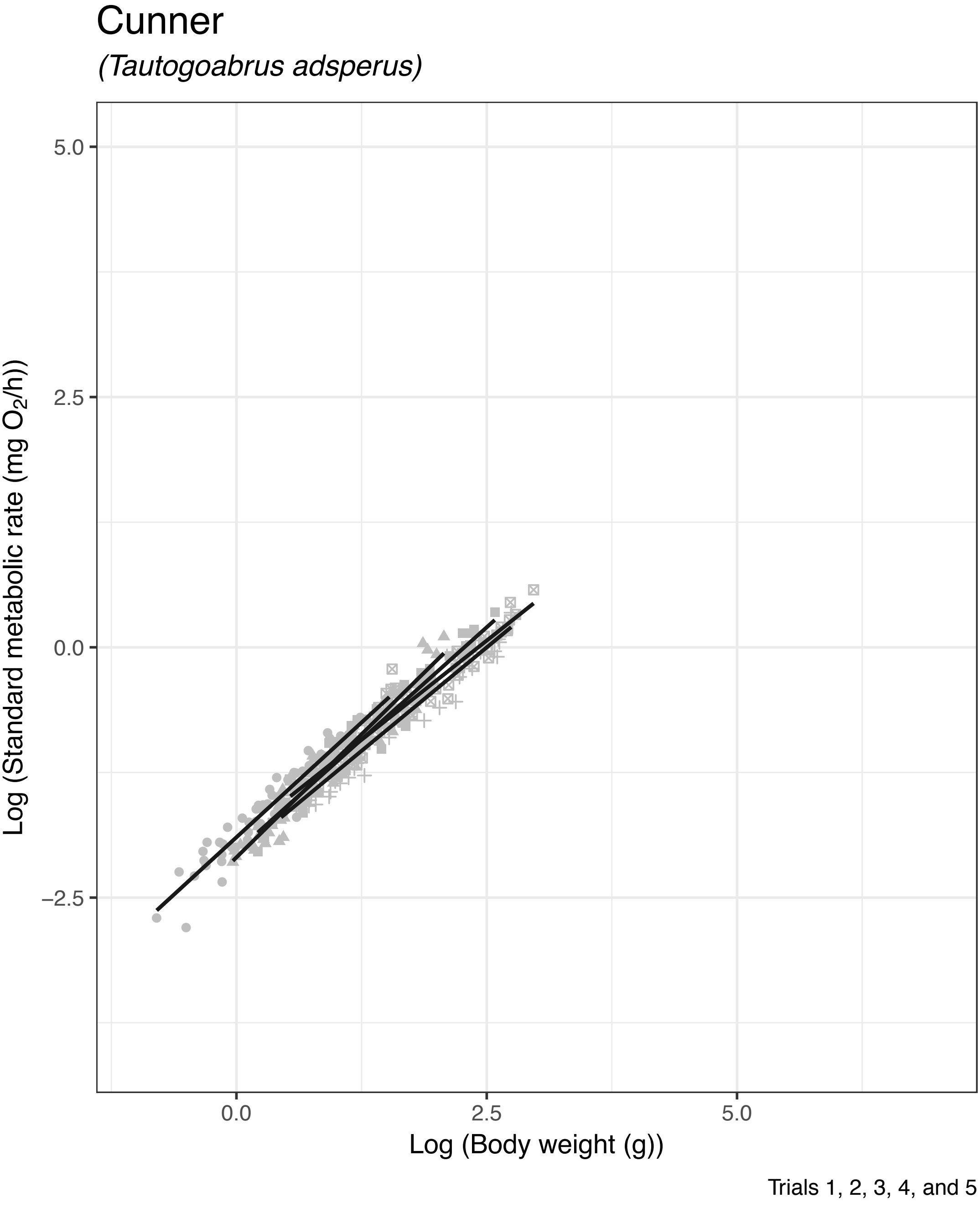
Cunner
(Tautogoabrus adsperus)
5.0
2.5
0.0
−2.5
Log (Standard metabolic rate (mg O2/h))
0.0
2.5
5.0
Log (Body weight (g))
Trials 1, 2, 3, 4, and 5

European Eel

(Anguilla anguilla)

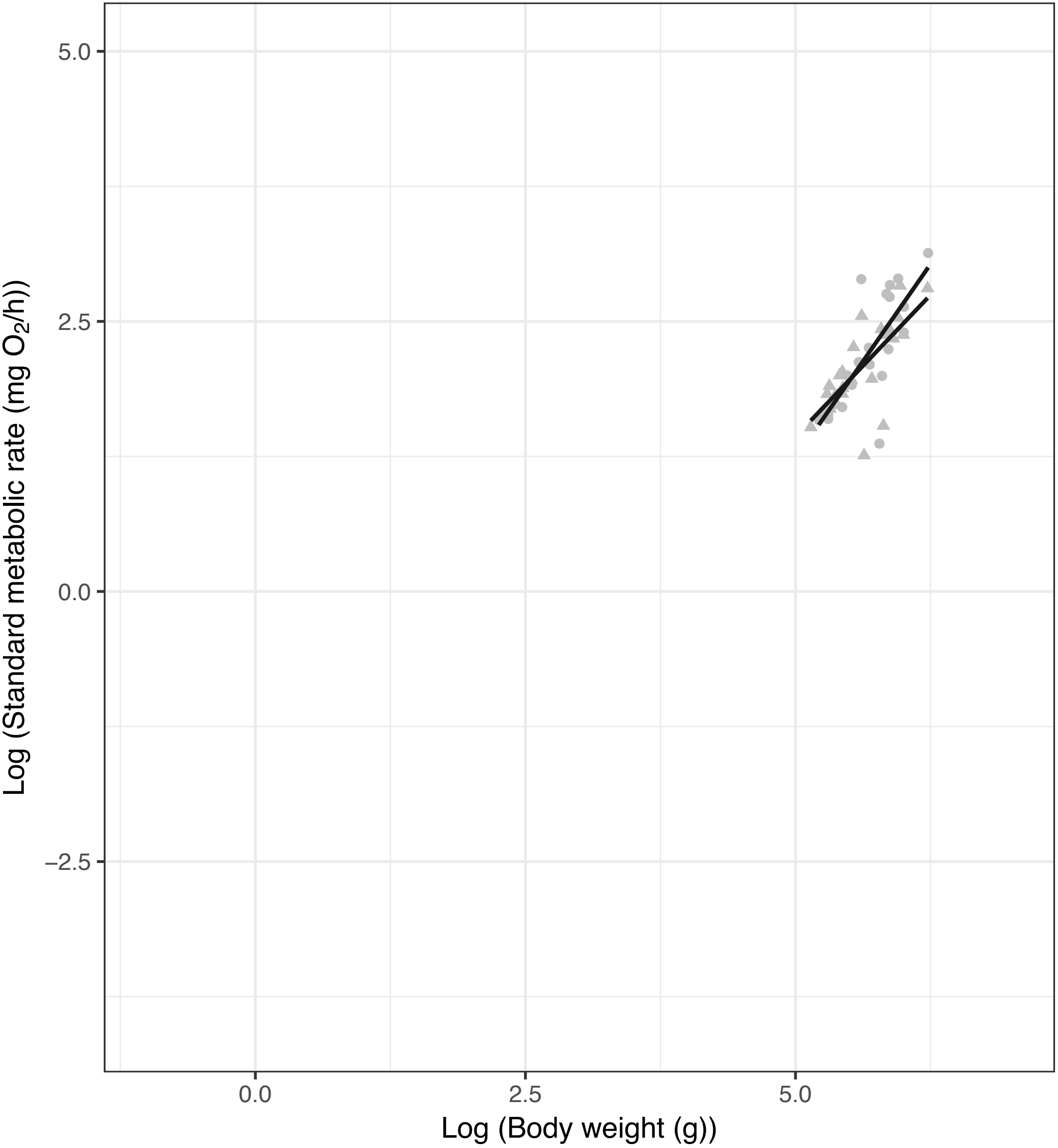

Trial 13 and 14

Hapuku Wreckfish

(Polyprion oxygeneios)

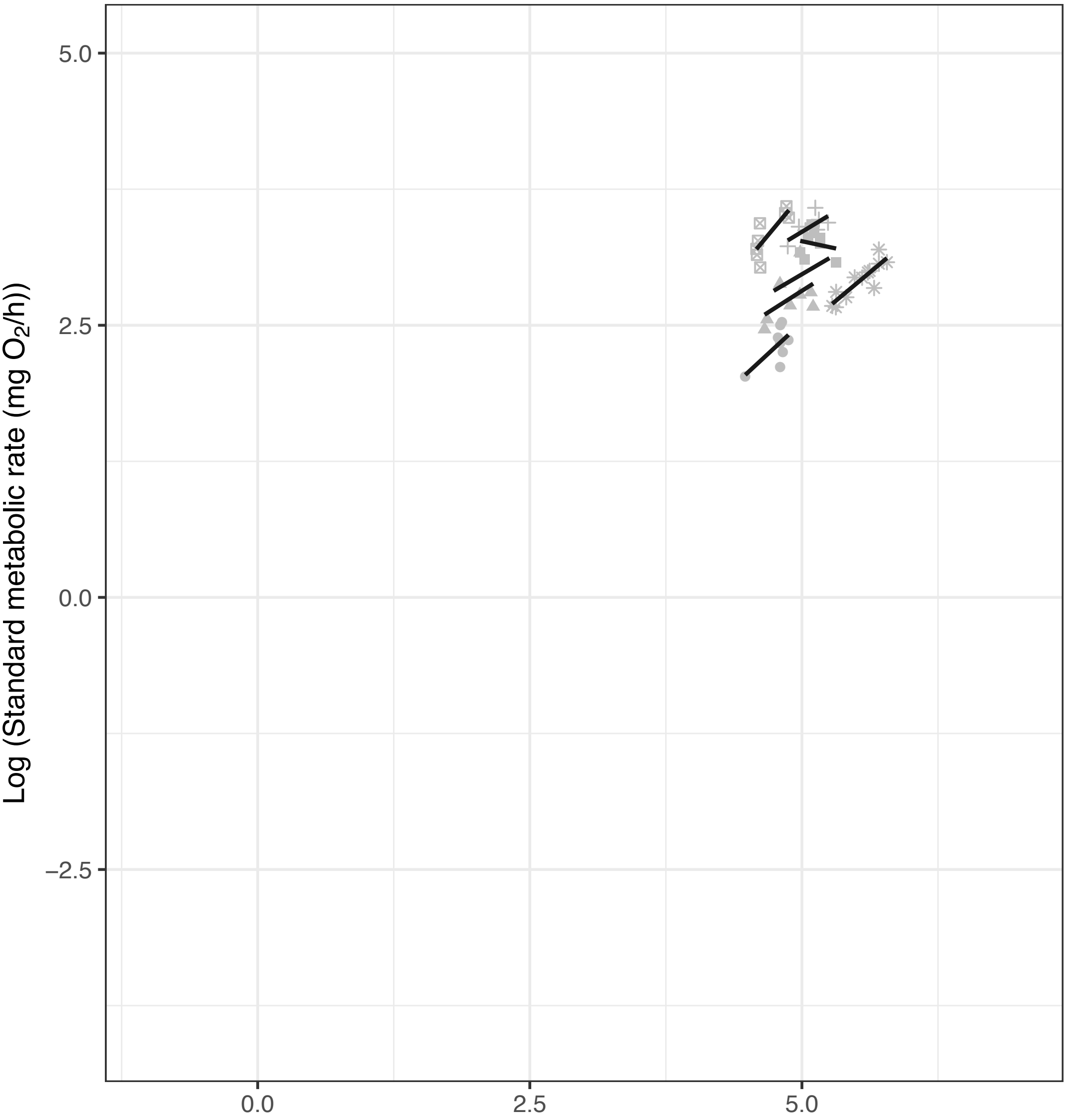

Trials 26, 27, 28, 29, 30, 33, and 34

Polar Cod

(Boreogadus saida)

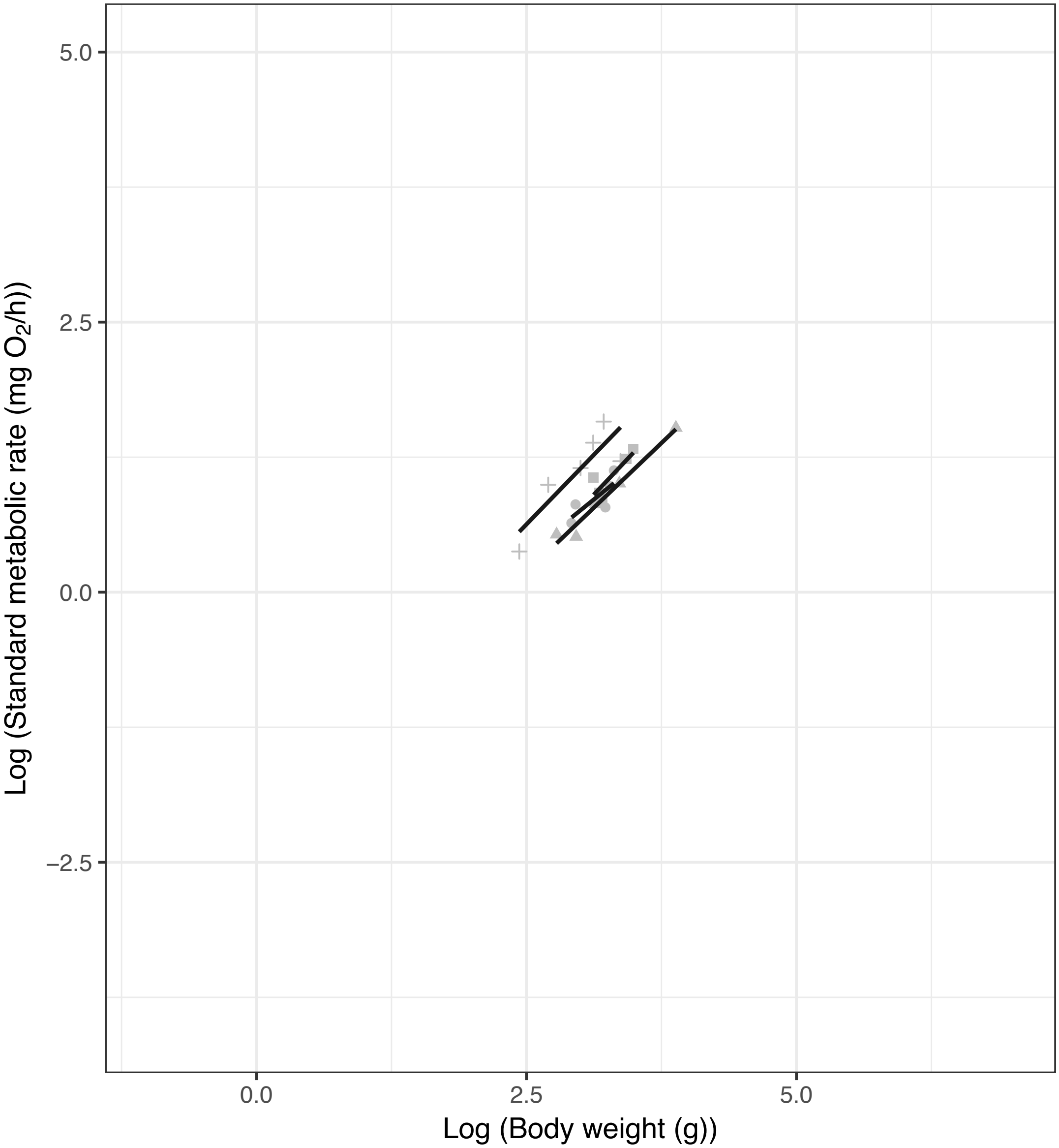

Trials 15, 16, 17, and 18

Blue−green Puller

(Chromis viridis)

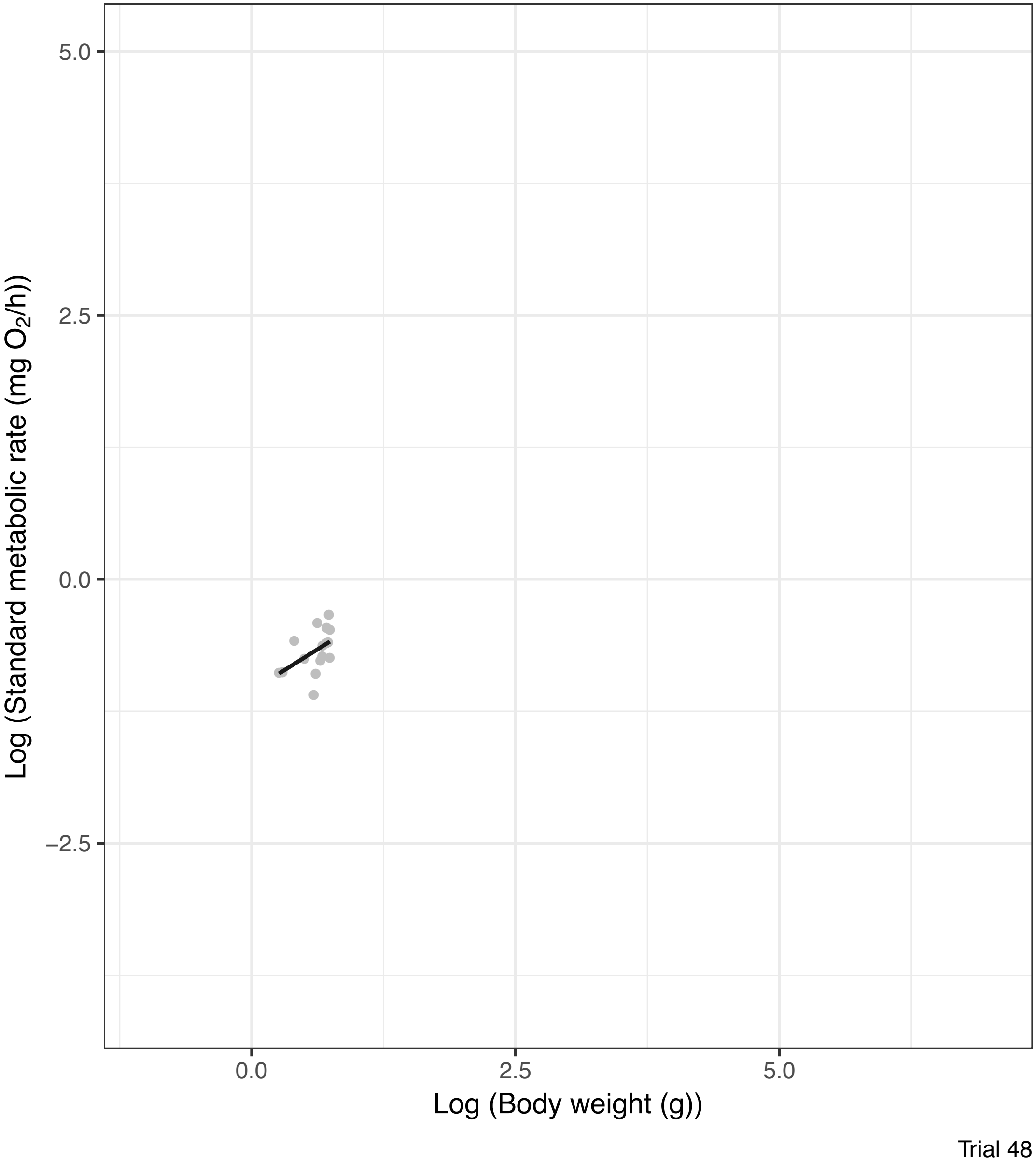

Trial 48

Rainbow Trout

(Oncorhynchus mykiss)

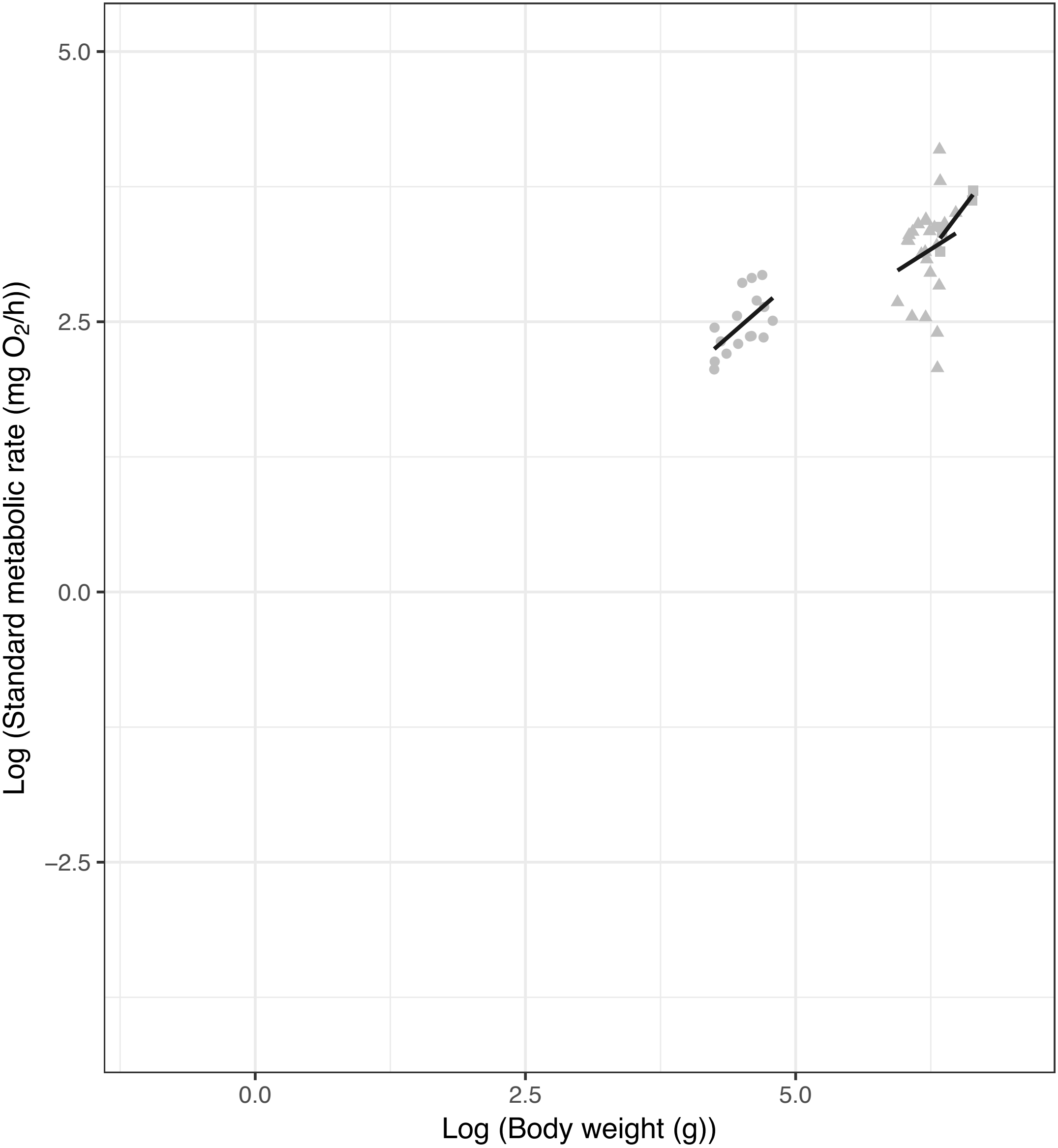

Trials 31, 41, and 42

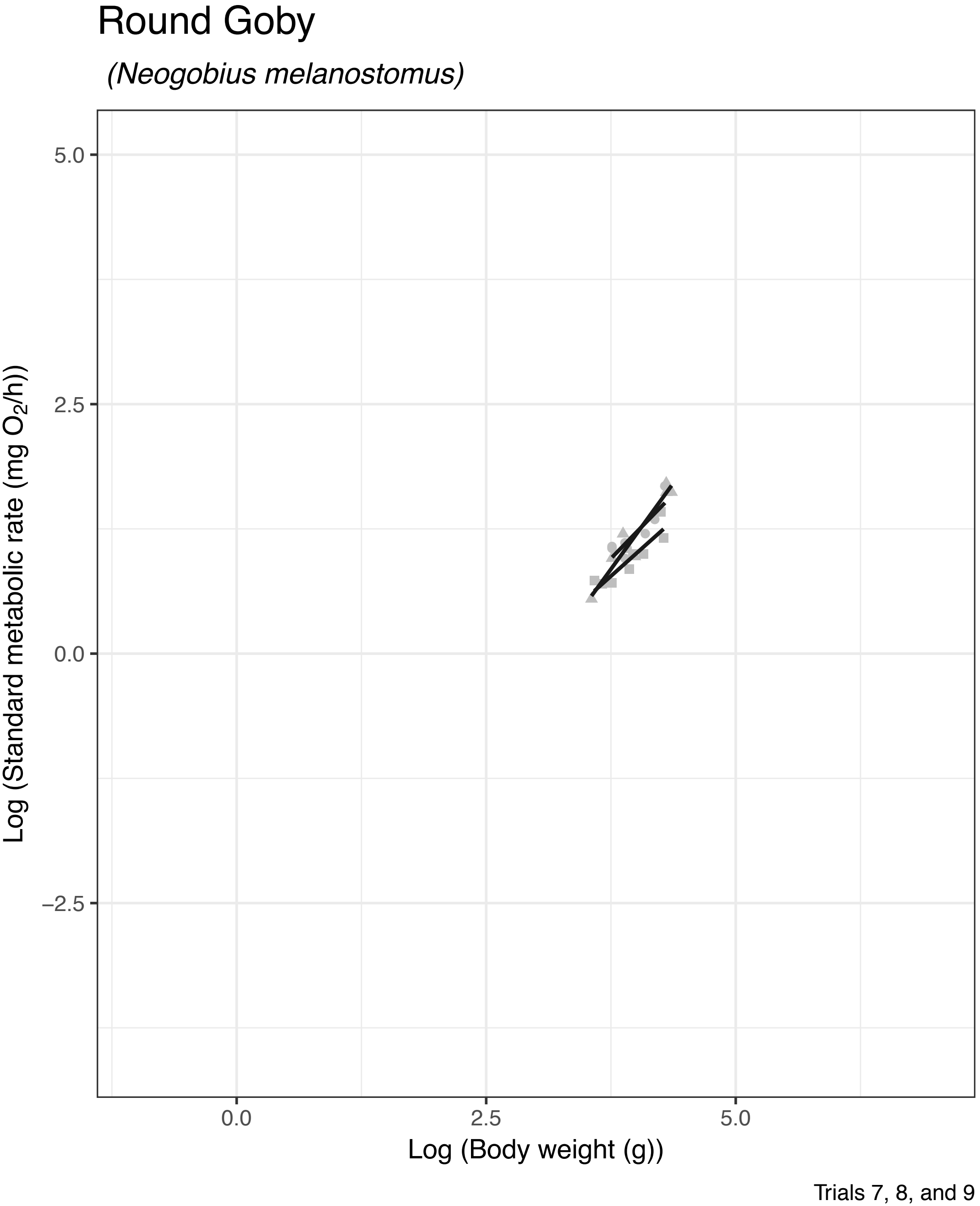
Round Goby
(Neogobius melanostomus)
5.0
2.5
0.0
−2.5
Log (Standard metabolic rate (mg O2/h))
0.0
2.5
5.0
Log (Body weight (g))
Trials 7, 8, and 9

European Sea Bass

(Dicentrarchus labrax)

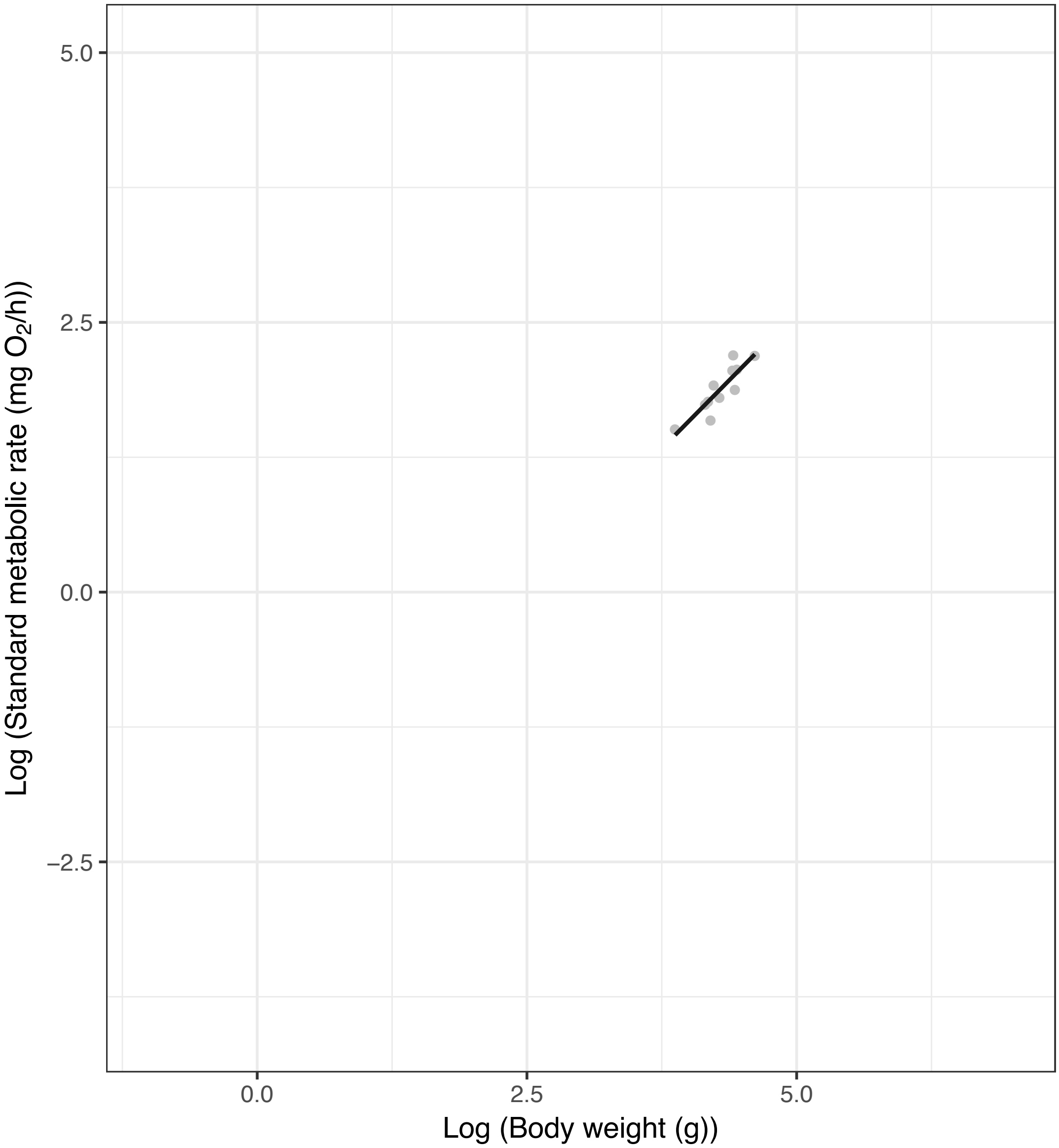

Trial 54

Three−spined Stickleback

(Gasterosteus aculeatus)

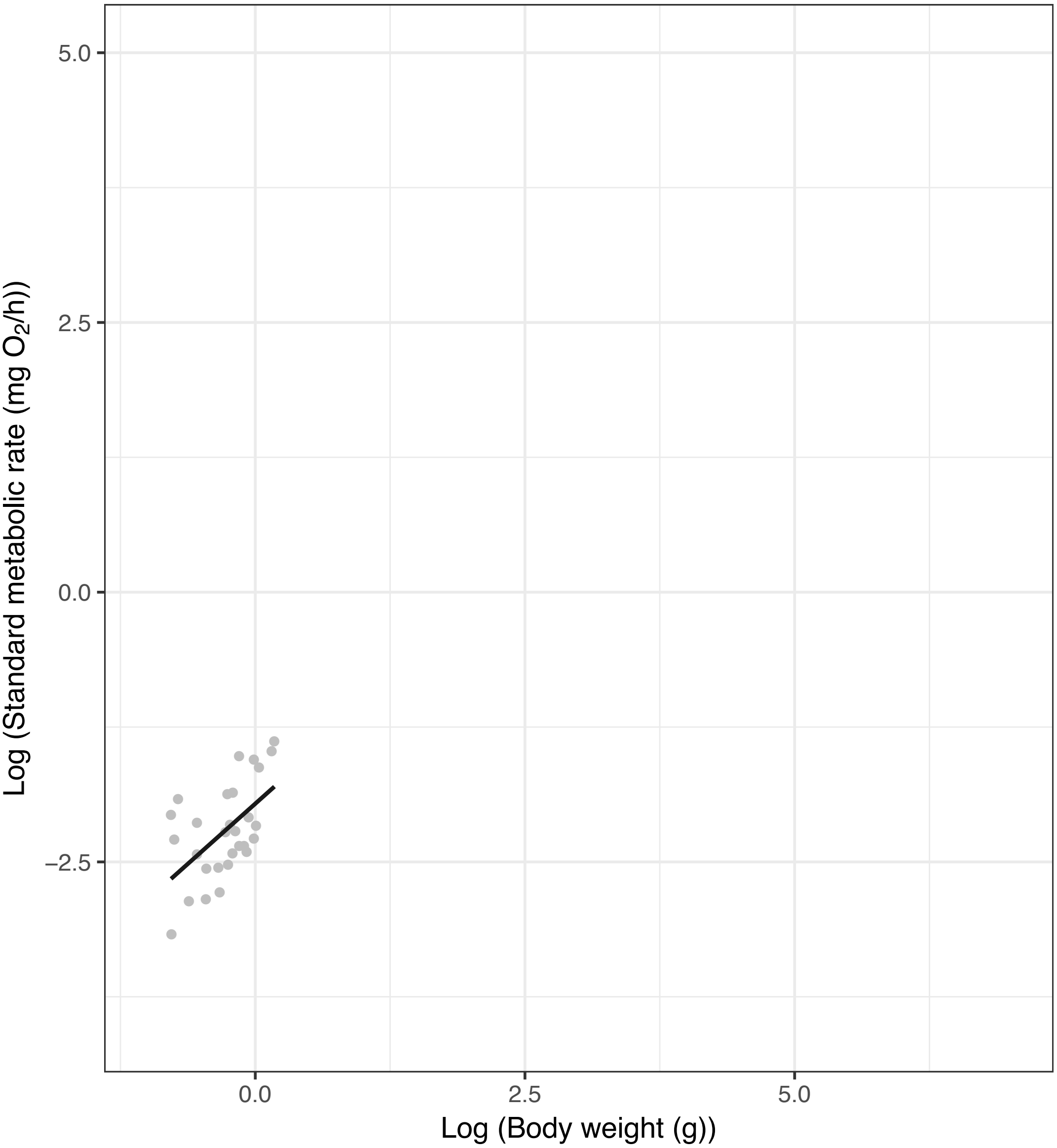

Trial 35

Twister

(Bellapiscis medius)

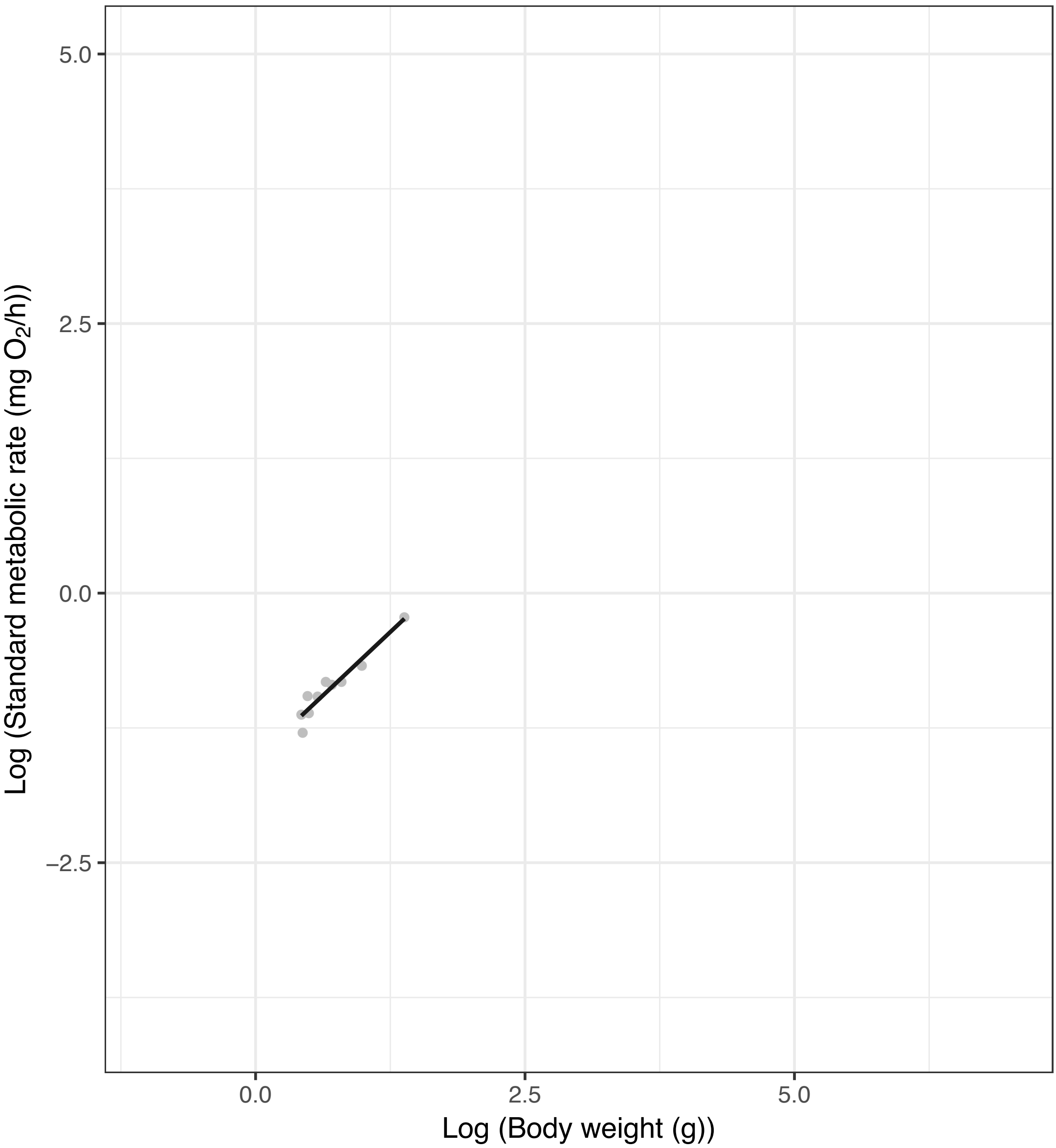

Trial 39

Supplementary material 2: R output of each model with a free parameter for the slope

Model Suite 1: Random Intercept models
Fixed effect: Weight, Temp; Random effect: Species; Nested effect: Trial

Formula: log(smr) ~ log(bw_g) + temp + (1 | species) + (1 | species:trial)

AIC	BIC	logLik	deviance
-135.8	-104.1	73.9	-147.8

Random effects:

Groups	Name	Variance	Std.Dev.
species:trial	(Intercept)	0.02729	0.1652
species	(Intercept)	0.19473	0.4413
Residual		0.04673	0.2162

Fixed effects:

	Estimate	Std. Error	t value
(Intercept)	-2.976927	0.189428	-15.715
log(bw_g)	0.868078	0.014608	59.423
temp	0.065893	0.008853	7.443

Model Suite 2: Estimated species intercept models
Fixed effect: Weight, Temp & Species, Nested effect: Trial

Formula: log(smr) ~ log(bw_g) + temp + species + (1 | species:trial) - 1

AIC	BIC	logLik	deviance
-175.5	-69.8	107.7	-215.5

Random effects:

Groups	Name	Variance	Std.Dev.
species:trial	(Intercept)	0.01764	0.1328
Residual		0.04672	0.2161

Fixed effects:

	Estimate	Std. Error	t value
log(bw_g)	0.866399	0.014747	58.749
temp	0.079591	0.008419	9.453
speciesAtlantic_Cod	-2.595677	0.128265	-20.237
speciesAtlantic_Salmon	-2.967810	0.156093	-19.013
speciesBarramundi	-3.845463	0.248164	-15.496
speciesBlue_green_puller	-3.506407	0.283329	-12.376
speciesBrown_Trout	-3.088307	0.143155	-21.573
speciesCommon_Minnow	-3.221242	0.139432	-23.103
speciesCommon_Triplefin	-3.383674	0.173440	-19.509
speciesCunner	-3.201778	0.141166	-22.681
speciesE_sea_bass	-3.152090	0.212755	-14.816
speciesEuropean_Eel	-4.347451	0.212546	-20.454
speciesHapuku_Wreckfish	-2.793287	0.178356	-15.661
speciesPolar_Cod	-2.087451	0.100664	-20.737
speciesRainbow_Trout	-2.974013	0.163135	-18.230
speciesRound_Goby	-3.768955	0.185149	-20.356
speciesThree_Spine_Stickleback	-3.050021	0.171394	-17.795
speciesTwister	-3.156603	0.231724	-13.622

Model Suite 3: Random intercepts with random slopes
Fixed Effect: Weight & Temp; Random: Species & Slope; Nested effect: Trial

Formula: log(smr) ~ log(bw_g) + (1 + log(bw_g) | species:trial) + temp + (1 | species)

AIC	BIC	logLik	deviance
-147.8	-105.6	81.9	-163.8

Random effects:

Groups	Name	Variance	Std.Dev.
species:trial	(Intercept)	0.070458	0.2654
log(bw_g)		0.004998	0.0707
species	(Intercept)	0.231253	0.4809
Residual		0.045650	0.2137

Fixed effects:

	Estimate	Std. Error	t value
(Intercept)	-3.019453	0.206274	-14.638
log(bw_g)	0.892427	0.021353	41.794
temp	0.064432	0.009122	7.063

Model Suite 4: Estimated species intercept models with random slopes
Fixed Effect: Weight, Temp & Species; Random: Slope; Nested effect: Trial

Formula: log(smr) ~ log(bw_g) + (0 + log(bw_g) | species:trial) + temp + species + (1 | species:trial) - 1

AIC	BIC	logLik	deviance
-173.6	-62.6	107.8	-215.6

Random effects:

Groups	Name	Variance	Std.Dev.
species.trial	log(bw_g)	9.646e-05	0.009821
species.trial.1	(Intercept)	1.690e-02	0.130014
Residual		4.664e-02	0.215967

Fixed effects:

	Estimate	Std. Error	t value
log(bw_g)	0.86787	0.01503	57.737
temp	0.07951	0.00860	9.245
speciesAtlantic_Cod	-2.60234	0.13075	-19.904
speciesAtlantic_Salmon	-2.97524	0.15883	-18.732
speciesBarramundi	-3.85023	0.25339	-15.195
speciesBlue_green_puller	-3.50494	0.28662	-12.229
speciesBrown_Trout	-3.09260	0.14562	-21.238
speciesCommon_Minnow	-3.21739	0.13966	-23.037
speciesCommon_Triplefin	-3.38329	0.17603	-19.220
speciesCunner	-3.20142	0.14308	-22.374
speciesE_sea_bass	-3.15763	0.21737	-14.527
speciesEuropean_Eel	-4.36195	0.21847	-19.966
speciesHapuku_Wreckfish	-2.79732	0.18231	-15.344
speciesPolar_Cod	-2.09188	0.10157	-20.596
speciesRainbow_Trout	-2.97451	0.16811	-17.694
speciesRound_Goby	-3.77453	0.18875	-19.998
speciesThree_Spine_Stickleback	-3.04785	0.17055	-17.871
speciesTwister	-3.15609	0.23313	-13.538

Supplementary material 3: Lobster repeated body mass (grams) measurement data used in Box 1 (NC=Not conducted)

ID	weight1 (g)	weight2 (g)	weight3 (g)	Weight double dry time (g)
1	930	937	938	932
2	2725	2739	2744	2725
3	818	820	822	NC
4	1214	1222	1226	NC
5	2430	2432	2437	2426
6	231	233	230	227
7	240	243	242	NC
8	179	178	178	175
9	184	184	185	NC
10	1859	1865	1859	NC
11	915	917	919	912
12	242	243	242	NC
13	539	539	539	535
14	385	382	392	380
15	491	492	492	NC
16	436	435	439	NC
17	616	618	618	NC
22	599	601	602	NC
23	432	432	432	NC
24	497	496	499	NC
25	532	534	533	NC
26	195	195	195	NC
27	288	289	288	NC
28	1382	1391	1388	NC
29	188	188	189	NC
31	433	433	435	NC
36	386	387	388	NC
37	334	333	335	NC
38	189	189	189	NC
39	716	715	717	NC
40	466	465	468	NC
41	1112	1116	1119	NC

42	1167	1171	1171	NC
43	767	767	767	NC
44	328	328	329	NC
45	411	412	412	NC
46	420	419	418	NC
47	210	209	211	NC
48	291	290	290	NC
49	1578	1577	1578	NC
50	1212	1214	1214	NC
51	368	368	367	NC
52	411	413	413	NC
53	2575	2575	2576	NC
54	1289	1297	1293	NC

Supplementary material 4: Output of models described in Box 5 used to evaluate temperature

In the body of this paper (Box 5), for conceptual clarity, we have written the joint effects of log(weight) and the log(weight) x Temp interaction as a temperature sensitive coefficient for log(weight), $\hat{\beta}(\text{temp})$. $\hat{\beta}(\text{temp})$ is a linear combination of $\hat{\beta}_{\log(weight)}$ and $\hat{\beta}_{\log(weight)*temp}$, with a combination weight vector of (1 T). When estimated, $\hat{\beta}_{\log(weight)}$ and $\hat{\beta}_{\log(weight)*temp}$ are random variables—they are estimated with error. The variance of any linear combination of a random vector is given by a quadratic form of the variance/covariance matrix for the random vector (see Searle 1971, p 40).

In this case, the standard error for the temperature sensitive scaling is

$$SE\ \hat{\beta}(\text{Temp}) = \sqrt{var\left(\hat{\beta}_{\log(weight)}\right) + var\left(\hat{\beta}_{\log(weight)*temp}\right) * temp^2 + 2 * covar(\log(weight) * temp)}$$

where,

$var(\hat{\beta}_{\log(weight)})$ is the variance of the log(weight) parameter,

$var(\hat{\beta}_{\log(weight)*temp})$ is the variance of the log(weight)*temp interaction parameter,

and,

$covar(\hat{\beta}_{\log(weight)*temp})$ is the covariance of $\hat{\beta}_{\log(weight)}$ and $\hat{\beta}_{\log(weight)*temp}$. Values of parameter variance and covariance can be extracted from the lme4 package using the covar(model) command.

Temp interaction on the fixed slope model

No temp
M17: log(smr) ~ log(bw_g) + (1 | species) + (1 | species:trial)

Fixed factor temp
M1: log(smr) ~ log(bw_g) + temp + (1 | species) + (1 | species:trial)

Fixed factor temp with interaction
M18: log(smr) ~ log(bw_g) + temp + temp * (log(bw_g)) + (1 | species)

Temp interaction on the random slope model

No temp
M19: log(smr) ~ log(bw_g) + (1 + log(bw_g) | species:trial) + (1 |species)

Fixed factor temp
M9: log(smr) ~ log(bw_g) + (1 + log(bw_g) | species:trial) + temp + (1 | species)

Fixed factor temp with interaction
M20:log(smr) ~ log(bw_g) + (1 + log(bw_g) | species:trial) + temp + temp * (log(bw_g)) + (1 |species) + (1 | species:trial)

OUTPUT

M17: Formula: log(smr) ~ log(bw_g) + (1 | species) + (1 | species:trial)

AIC	BIC	logLik	deviance	df.resid
-107.2	-80.7	58.6	-117.2	1451

Random effects:

Groups	Name	Variance	Std.Dev.
species:trial	(Intercept)	0.07578	0.2753
species	(Intercept)	0.09193	0.3032
Residual		0.04679	0.2163

Fixed effects:

	Estimate	Std. Error	t value
(Intercept)	-1.91897	0.09988	-19.21
log(bw_g)	0.87241	0.01475	59.14

M1: Formula: log(smr) ~ log(bw_g) + temp + (1 | species) + (1 | species:trial)

AIC	BIC	logLik	deviance	df.resid
-135.9	-104.2	74.0	-147.9	1450

Random effects:

Groups	Name	Variance	Std.Dev.
species:trial	(Intercept)	0.02732	0.1653
species	(Intercept)	0.19460	0.4411
Residual		0.04673	0.2162

Fixed effects:

	Estimate	Std. Error	t value
(Intercept)	-2.976252	0.189430	-15.712
Log(bw_g)	0.868000	0.014608	59.420
temp	0.065868	0.008854	7.439

M18: Formula: log(smr) ~ log(bw_g) + temp + temp * (log(bw_g)) + (1 | species) + (1 | species:trial)

AIC	BIC	logLik	deviance	df.resid
-134.6	-97.7	74.3	-148.6	1449

Random effects:

Groups	Name	Variance	Std.Dev.
species:trial	(Intercept)	0.02800	0.1673
species	(Intercept)	0.19842	0.4454
Residual		0.04666	0.2160

Fixed effects:

	Estimate	Std. Error	t value
(Intercept)	-2.830956	0.254823	-11.110
log(bw_g)	0.828807	0.047818	17.333
temp	0.056580	0.013960	4.053
log(bw_g):temp	0.002536	0.002931	0.865

Variance-Covariance Matrix

	(Intercept)	log(bw_g)	temp	log(bw_g):temp
(Intercept)	0.0649346847	-0.0083274299	-3.110329e-03	4.935793e-04

log(bw_g)	-0.0083274299	0.0022865299	4.885162e-04	-1.334289e-04
temp	-0.0031103293	0.0004885162	1.948952e-04	-3.141428e-05
log(bw_g):temp	0.0004935793	-0.0001334289	-3.141428e-05	8.589476e-06

M19: Formula: log(smr) ~ log(bw_g) + (1 + log(bw_g) | species:trial) + (1 | species)

AIC	BIC	logLik	deviance	df.resid
-123.1	-86.1	68.5	-137.1	1449

Random effects:

Groups	Name	Variance	Std.Dev.	Corr
species:trial	(Intercept)	0.11710	0.3422	
	log(bw_g)	0.01064	0.1031	-0.80
species	(Intercept)	0.13715	0.3703	
Residual		0.04538	0.2130	

Fixed effects:

	Estimate	Std. Error	t value
(Intercept)	-2.03282	0.13165	-15.44
log(bw_g)	0.90837	0.02582	35.18

M9: Formula: log(smr) ~ log(bw_g) + (1 + log(bw_g) | species:trial) + temp + (1 | species)

AIC	BIC	logLik	deviance	df.resid
-147.8	-105.6	81.9	-163.8	1448

Random effects:

Groups	Name	Variance	Std.Dev.	Corr
species:trial	(Intercept)	0.070458	0.2654	
	log(bw_g)	0.004998	0.0707	-0.86
species	(Intercept)	0.231253	0.4809	
Residual		0.045650	0.2137	

Fixed effects:

	Estimate	Std. Error	t value
(Intercept)	-3.019453	0.206274	-14.638
log(bw_g)	0.892427	0.021353	41.794
temp	0.064432	0.009122	7.063

M20: Formula: log(smr) ~ log(bw_g) + (1 + log(bw_g) | species:trial) + temp + temp * (log(bw_g)) + (1 | species)

AIC	BIC	logLik	deviance	df.resid
-145.9	-98.4	82.0	-163.9	1447

Random effects:

Groups	Name	Variance	Std.Dev.	Corr
species:trial	(Intercept)	0.070663	0.2658	
	log(bw_g)	0.004901	0.0700	-0.86
species	(Intercept)	0.232109	0.4818	
Residual		0.045649	0.2137	

Fixed effects:

	Estimate	Std. Error	t value
(Intercept)	-2.958265	0.281762	-10.499
log(bw_g)	0.874656	0.057701	15.158
temp	0.060812	0.014717	4.132
log(bw_g):temp	0.001061	0.003270	0.324

Variance-Covariance Matrix

	(Intercept)	log(bw_g)	temp	log(bw_g):temp

(Intercept)	0.0793900196	-0.0117618489	-3.573021e-03	6.271892e-04
log(bw_g)	-0.0117618489	0.0033294100	6.198137e-04	-1.753920e-04
temp	-0.0035730207	0.0006198137	2.165976e-04	-3.776815e-05
log(bw_g):temp	0.0006271892	-0.0001753920	-3.776815e-05	1.069525e-05
